# The impact of pre-, pro- and synbiotics supplementation in colorectal cancer treatment: a systematic review

**DOI:** 10.3389/fonc.2024.1395966

**Published:** 2024-05-14

**Authors:** Mariana Melo Moreira, Marta Carriço, Manuel Luís Capelas, Nuno Pimenta, Teresa Santos, Susana Ganhão-Arranhado, Antti Mäkitie, Paula Ravasco

**Affiliations:** ^1^ Universidade Católica Portuguesa, Faculty of Health Sciences and Nursing (FCSE), Lisboa, Portugal; ^2^ Champalimaud Foundation, Nutrition Service of Champalimaud Clinical Center, Lisbon, Portugal; ^3^ Universidade Católica Portuguesa, Center for Interdisciplinary Research in Health (CIIS), Lisbon, Portugal; ^4^ Polytechnic Institute of Santarém, Sport Sciences School of Rio Maior, Rio Maior, Portugal; ^5^ Sport Physical Activity and Health Research and Innovation Center (SPRINT), Santarém Polytechnic University, Rio Maior, Portugal; ^6^ Faculdade de Ciências Sociais e Tecnologia, Universidade Europeia de Lisboa, Lisbon, Portugal; ^7^ Atlântica, Instituto Universitário, Barcarena, Portugal; ^8^ CINTESIS, Centre for Health Technology and Services Research, Porto, Portugal; ^9^ Department of Otorhinolaryngology-Head and Neck Surgery, Helsinki University Hospital and University of Helsinki, Helsinki, Finland; ^10^ Research Program in Systems Oncology, Faculty of Medicine, University of Helsinki, Helsinki, Finland; ^11^ Division of Ear, Nose and Throat Diseases, Department of Clinical Sciences, Intervention and Technology, Karolinska Institute and Karolinska University Hospital, Stockholm, Sweden; ^12^ Universidade Católica Portuguesa, Católica Medical School, Rio de Mouro, Portugal; ^13^ Center for Interdisciplinary Research Egas Moniz, Egas Moniz School of Health & Science, Almada, Portugal

**Keywords:** probiotics, synbiotics, prebiotics, microbiota, colorectal neoplasms, systematic review

## Abstract

**Introduction:**

The effectiveness of the supplementation of prebiotics, probiotics and synbiotics as a therapeutic approach in colorectal cancer (CRC) remains unclear. The aim of this systematic review is to critically examine the current scientific evidence on the impact of modulating the microbiota, through the use of prebiotics, probiotics and synbiotics, in patients diagnosed with CRC undergoing treatment, to determine the potential therapeutic use of this approach.

**Methods:**

This systematic review was made according to the PRISMA 2020 guidelines. Inclusion criteria were randomized controlled trials (RCT) comparing the impact of pre-, pro-, or synbiotic supplementation with placebo or standard care in patients with CRC undergoing treatment. Exclusion criteria were non-human studies, non-RCTs, and studies in languages other than English or Portuguese. Six databases were consulted, namely, Cochrane Library, Pubmed, Scopus, Cinahl, MedicLatina and Web of Science until May of 2023. RAYYAN software was used to manage the search results and risk of bias was assessed according to the guidelines of the Cochrane Collaboration using the Rob 2.0 tool.

**Results:**

Twenty-four RCTs met the inclusion criteria and were included in this review. Administration of pre-, pro-, or synbiotics improved surgical outcomes such as the incidence of infectious and non-infectious postoperative complications, return to normal gut function, hospital length of stay, and antibiotic usage. The supplementation of these microorganisms also alleviated some symptoms from chemotherapy and radiotherapy, mainly diarrhea. Evidence on the best approach in terms of types of strains, dosage and duration of intervention is still scarce.

**Conclusions:**

Pre-, pro-, and synbiotics supplementation appears to be a beneficial therapeutic approach in CRC treatment to improve surgical outcomes and to alleviate side-effects such as treatment toxicity. More RCTs with larger sample sizes and less heterogeneity are needed to confirm these potential benefits and to determine the best strains, dosage, and duration of administration in each situation.

**Systematic review registration:**

https://www.crd.york.ac.uk/prospero, identifier CRD42023413958.

## 1 Introduction

According to GLOBOCAN, in 2022, colorectal cancer (CRC) ranked as the third most diagnosed cancer, with over 1.9 million new cases, and the second most deadly malignancy causing roughly 904,000 deaths. This accounted for 9.3% of cancer-related deaths (1). GLOBOCAN also estimates that by 2040 the burden of CRC will rise to 3.2 million new cases and 1.6 million deaths with most cases predicted to occur in developing countries with the numbers increasing along with the increase of the Human Development Index. Conversely, in highly developed countries, where the screening is now a routine, numbers are expected to stabilize or even decline (2, 3).

Surgery stands as the primary treatment for CRC, but chemotherapy and radiotherapy are also commonly used as neoadjuvant or adjuvant treatments. These approaches often lead to several side-effects such as postoperative infectious complications, diarrhea, vomiting, nausea, etc. (4–6). More recently, immunotherapy and targeted therapy have emerged as viable options in select cases (5, 7).

The etiology of CRC is multifactorial, involving genetic factors, epigenetic alterations, and environmental factors such as being overweight, smoker, heavy drinker and following an unhealthy diet (8–10). More recently, the development of CRC has also been associated with chronic inflammation, immune system dysfunction, and dysbiosis. Dysbiosis is the compositional and functional alteration caused by an imbalance between symbiotic and opportunistic microbiomes. It can be categorized in three types: loss of beneficial microbes, expansion of pathogenic microbes, and loss of microbial diversity (11, 12).

Over the past decade, the relationship between the gut microbiota and CRC has gained significant attention with studies showing that patients with CRC harbor a distinct microbiota composition compared to healthy control subjects (3, 13). These studies show that CRC patients’ microbiota has lower bacterial diversity, lower abundance of commensal bacteria such as *Akkermansia muciniphila*, *Lactobacillus rhamnosus* and *Bifidobacterium breve*, and higher abundance of pro-carcinogenic bacteria such as *Fusobacterium nucleatum, Escherichia coli, Bacteroides fragilis, Streptococcus gallolyticus* and *Peptostreptococcus anaerobius* (3, 10, 13, 14). Studies have also shown that butyrate-producing bacteria are less represented in CRC patients. Butyrate is a short-chain fatty acid with very important health-promoting and antineoplastic properties such as being the main energy source for colonocytes, maintaining the mucosal barrier integrity, reducing pro-inflammatory cytokines, and inducing apoptosis (9, 15, 16).

The microbiota has been studied not only as a potential risk factor for CRC but also as a therapeutic approach in the treatment of this malignancy through its modulation with pre-, pro-, and synbiotics (17, 18). Prebiotics are defined as a non-digestible food ingredient that promote changes in the composition and/or activity of the microbiota conferring health benefits to the host (3). On the other hand, according to the most accepted definition and the one proposed by the expert panel convened by the International Scientific Association of Probiotics and Prebiotics in 2014, probiotics are “live microorganisms which, when administered in an adequate amount, confer a health benefit to the host” (19). The most commonly used strains are from the *Lactobacillus, Bifidobacterium, Streptococcus* and *Enterococcus* genera (10, 14). When prebiotics and probiotics are administered together, in a way that prebiotics promote the growth and survival of probiotics, it’s called a synbiotic (20, 21).

Recent studies indicate that modulating the microbiota, through the supplementation of pre-, pro-, or synbiotics, appears to have an impact on CRC treatment. This can be due to the reduction of postoperative infectious and non-infectious complications and side-effects of chemotherapy and radiotherapy or even directly on the efficacy of the drugs used in chemotherapy or, more recently, on the sensitivity to immunotherapy (12, 22, 23).

While some systematic reviews have delved into this area (24–31), the majority focused on only one treatment for CRC, or one outcome and some results appear to be contradictory. To overcome these previous limitations, the aim of this systematic review is to critically examine the present scientific evidence, including more recent findings, on the impact of modulating the microbiota, through the use of pre-, pro-, and synbiotics, in CRC patients undergoing treatment. This is also performed to determine the potential therapeutic use of this approach.

## 2 Materials and methods

This systematic review followed the Preferred Reporting Items for Systematic Reviews and Meta-analyses (PRISMA) guidelines (32) and was registered in the PROSPERO database (registration number CRD42023413958).

### 2.1 Literature search

This systematic review was conducted in six databases, namely, Cochrane Library, Pubmed, Scopus, Cinahl, MedicLatina and Web of Science, until May 2023. The MESH terms or equivalents and search terms for title and abstract were selected according to the Population, Intervention, Comparison, Outcome and Study (PICOS) model. The MESH terms used for the Pubmed database are shown in **Table 1**. The search strategy for the databases is shown in detail in **Appendix 1**. The results were filtered to identify studies in the English or Portuguese language. The references of the selected studies were also scanned to identify additional studies missed in the initial search.

**Table 1:** Mesh terms used for research in Pubmed database.

	Mesh Terms
**Population**	**Colonic Neoplasms OR Colorectal Neoplasms OR Rectal Neoplasms OR Anus Neoplasms OR Colorectal Neoplasms, Hereditary Nonpolyposis OR Sigmoid Neoplasms OR Colitis-Associated Neoplasms**
**Intervention**	**Prebiotics OR Probiotics OR Synbiotics OR *Lactobacillus* OR *Bifidobacterium***
**Control**	–
**Outcome**	**Radiotherapy OR Immunotherapy OR Immune Checkpoint Inhibitors OR Antineoplastic Agents OR Colorectal Surgery OR Postoperative Complications OR Surgical Wound Infection OR Diarrhea OR Nausea OR Postoperative Nausea and Vomiting OR Vomiting OR Signs and Symptoms, Digestive OR Mucositis OR Quality of Life OR Biomarkers OR Biomarkers, Tumor**

### 2.2 Inclusion and exclusion criteria

The inclusion criteria were based on the PICOS model. The selected population comprised patients diagnosed with CRC (colon cancer or rectal cancer), the intervention was the supplementation with pre-, pro-, or synbiotics. The considered control treatment was placebo or standard care, the primary outcomes were the impact of this intervention on the efficacy, toxicity, or side-effects of treatments such as chemotherapy, radiotherapy, surgery or immunotherapy and the studies selected were randomized controlled studies.

The exclusion criteria were studies in languages other than English or Portuguese, studies where the population were patients with other types of cancer and studies where the intervention wasn’t exclusively the supplementation of prebiotics, probiotics or synbiotics.

### 2.3 Study selection

The studies obtained from the initial search were uploaded to the RAYYAN software and were analyzed and selected by two independent reviewers (MM and MC). The articles were screened by title and abstract and then full text of relevant studies were retrieved and assessed based on the inclusion and exclusion criteria. Disagreements in study selections were resolved by discussion between the two reviewers.

### 2.4 Data extraction

Data such as author, publication year, participant, placebo, and intervention details and outcomes were extracted from articles considered eligible and compiled in a summary table.

### 2.5 Risk of bias assessment

The risk of bias of all included studies was assessed through the RoB 2.0 tool, according to the guidelines of the Cochrane Collaboration (33), using Review Manager Software (Revman Web 5.5 - online). Risk of bias was assessed in the following domains: random sequence generation (selection bias), allocation concealment (selection bias), blinding of participants and personnel (performance bias), blinding of outcome assessment (detection bias), incomplete outcome data (attrition bias), selective reporting (reporting bias) and other bias. The risk of bias was then classified as high, low, or unclear risk.

## 3 Results

### 3.1 Study selection

**Figure 1:**
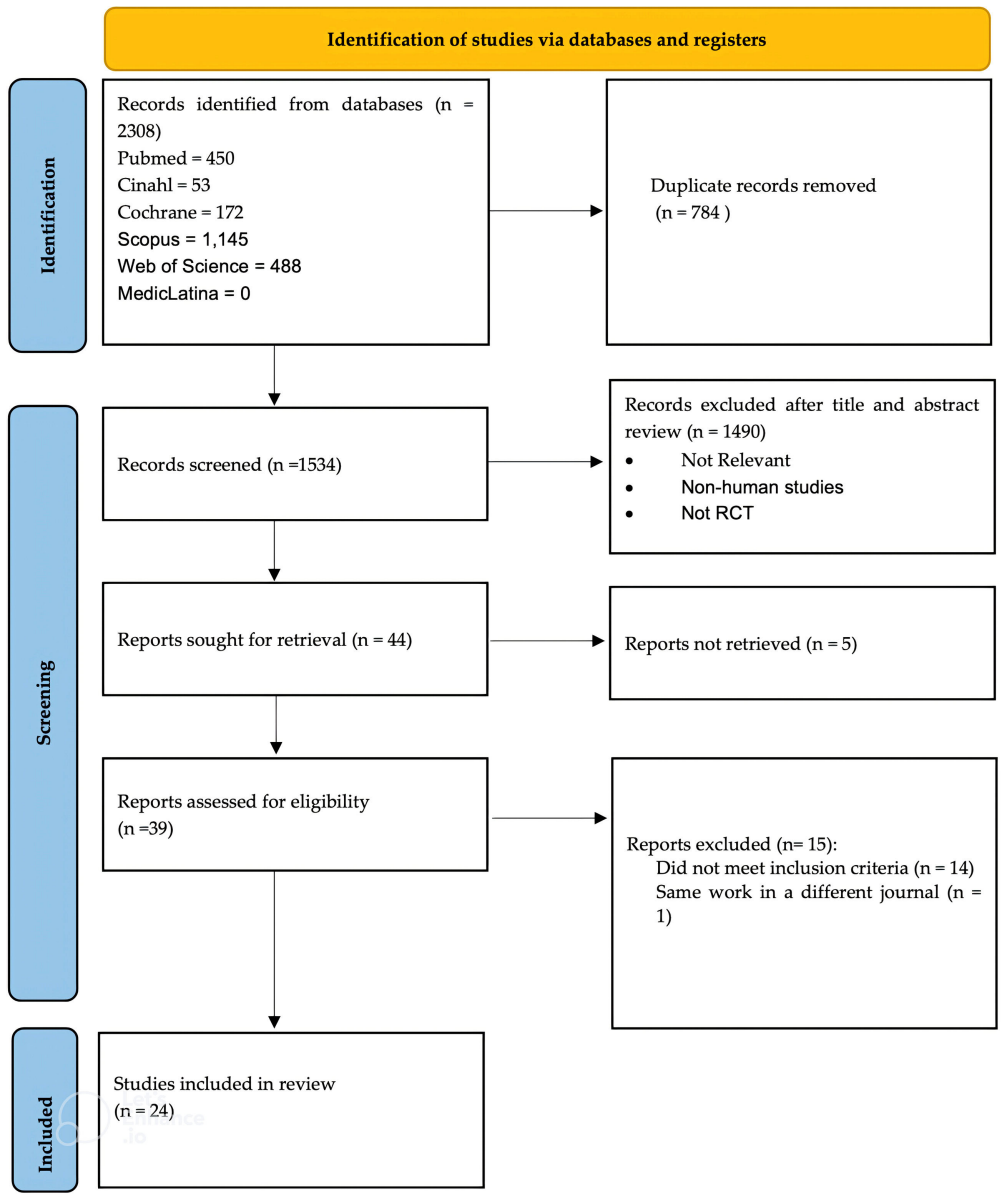
PRISMA flowchart that summarizes the screening and selection process.

A total of 2308 studies were obtained from the initial search through six databases, namely, Cochrane Library, Pubmed, Scopus, CINAHL, MedicLatina and Web of Science. These results were uploaded to the RAYYAN software, and the duplicates were identified (n = 784). After the duplicates were removed, 1524 articles remained and were screened by title and abstract. Afterwards, 1480 articles were excluded for reasons such as being non-human studies, being reviews or case reports or not being relevant to this review. The remaining 44 studies were full text screened for eligibility and 24 studies were included in this review. The other 20 studies were excluded because they did not meet the inclusion criteria or were unavailable in full-text format. The studies that did not meet the inclusion criteria are shown in **Appendix 2**. The PRISMA flow diagram, shown in **Figure 1**, summarizes the selection process.

### 3.2 Description of the selected studies

The 24 studies included in this review were all randomized controlled studies. The year of publication ranged from 2007 to 2023, where approximately 70% of the studies were published in the last 10 years. In terms of geographic localization the included studies are from China (n=6) (34–39), Turkey (n=2) (40, 41), Slovenia (n=2) (42, 43), Japan (n=2) (44, 45), Greece (n=2) (46, 47), Brazil (n=2) (48, 49), Iran (n=2) (50, 51), Malaysia (n=1) (52), Finland (n=1) (53), Sweden (n=1) (54), Slovakia (n=1) (55), Republic of Korea (n=1) (56) and Bosnia and Herzegovina (n=1) (57). There were a total of 2204 participants, 1139 in the intervention groups and 1065 in the control groups. Of these, 1220 participants were male, 984 were female, and their age ranged from 19 to 92 years. Considering the intervention, 11 out of the 24 studies used a mixture of probiotics (34–39, 46, 52, 55–57), 8 used synbiotics (42, 43, 45, 47–51), 3 used a single probiotic (44, 53, 54) and 2 studies used Kefir (40, 41). Species of the *Lactobacillus, Bifidobacterium* and *Enterococcus* genera were the most commonly used for the probiotics or synbiotics intervention. Placebo was used for the control group in 18 of the studies (34–42, 46–50, 52, 54–56) while the other 6 used standard care treatment (43–45, 51, 53, 57). Characterization of these studies is shown in **Table 2**.

**Table 2:** Characteristics of the 24 included studies.

Year	Author	Country	Study Design	Reference
2007	Österlund, P.	Finland	RCT	(53)
2008	Topuz, E.	Turkey	RCT	(40)
2009	Can, G.	Turkey	RCT	(41)
2010	Horvat, M.	Slovenia	RCT/double blind	(42)
2011	Liu, Z.	China	RCT/double blind	(34)
2012	Mangell, P.	Sweden	RCT	(54)
2012	Zhang, J.	China	RCT	(35)
2013	Liu, Z.	China	RCT/double blind	(36)
2014	Sadahiro, S.	Japan	RCT	(44)
2015	Kotzampassi, K.	Greece	RCT/double blind	(46)
2015	Mego, M.	Slovakia	RCT/double blind	(55)
2016	Komatsu, S.	Japan	RCT	(45)
2016	Tan, C.	Malaysia	RCT/double blind	(52)
2016	Krebs, B.	Slovenia	RCT/double blind	(43)
2016	Theodoropoulos, G.	Greece	RCT	(47)
2016	Yang, Y.	China	RCT	(37)
2017	Flesch, A.	Brazil	RCT/double blind	(48)
2019	Polakowski, C.	Brazil	RCT/double blind	(49)
2019	Xu, Q.	China	RCT	(38)
2019	Bajramagic, S.	Bosnia and Herzegovina	RCT	(57)
2020	Radvar, F.	Iran	RCT/double blind	(50)
2020	Park, I.	Korea	RCT/double blind	(56)
2023	Mohebian, F.	Iran	RCT	(51)
2023	Huang, F.	China	RCT	(39)

### 3.3 Risk of bias assessment

**Figure 2:**
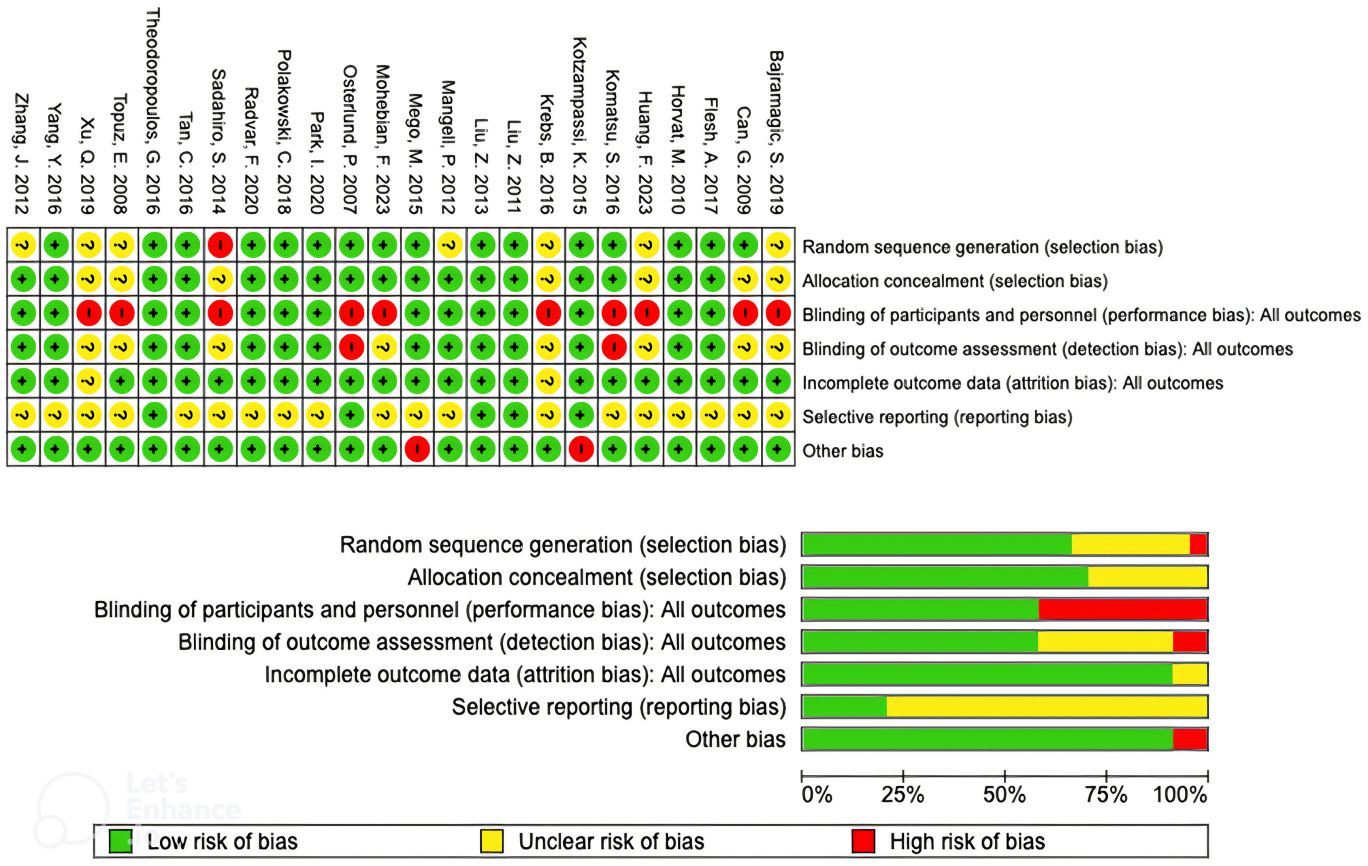
Risk of bias summary **(A)** and risk of bias assessment graph **(B)**.

The summary results of the risk-of-bias assessment are shown in **Figure 2**. All studies included in this review were randomized but 7 studies failed to mention how the randomization was done (35, 38–40, 43, 54, 57). Therefore, there is an unclear risk of bias in this parameter and in 1 study randomizations were performed by one of the authors leading to a high risk of bias (44). In terms of allocation concealment, 7 studies had an unclear risk of bias as there was no clear description if allocation was concealed until the beginning of the intervention (38–41, 43, 44, 57). Ten studies presented a high risk of bias in the performance domain because participants and/or personnel weren’t blinded during the intervention (38–41, 43–45, 51, 53, 57). Blinding of outcome assessment risk was unclear in 8 studies (38–41, 43, 44, 51, 57) and high in 2 studies (45, 53) while incomplete data outcome risk was unclear in only 2 of the studies (38, 43). Nineteen of the studies were considered to have an unclear risk in terms of selective reporting (35, 37–45, 48–52, 54–57) and 2 studies were considered to have a high risk of other bias because they were ended prematurely before full recruitment of participants was completed (46, 55).

### 3.4 Outcomes

Out of the 24 included studies, 17 evaluated the impact of probiotics or synbiotics supplementation in colorectal cancer surgery (34–38, 42–49, 52, 54, 56, 57) while the remaining 7 studied the impact on treatment with chemotherapy (39–41, 50, 51, 53, 55). One study selected patients undergoing chemoradiotherapy so outcomes on the impact of this approach in treatment with radiotherapy was also assessed (50). None of the studies evaluated the impact of probiotics or synbiotics supplementation in CRC patients undergoing immunotherapy.

#### 3.4.1 Surgery

The impact of probiotics or synbiotics supplementation in CRC patients undergoing surgery treatment was assessed in 17 studies (34–38, 42–49, 52, 54, 56, 57). Details from each study are shown in **Table 3**. Six studies used synbiotics for the intervention (42, 43, 45, 47–49), 9 used a mixture of probiotics strains (34–38, 46, 52, 56, 57) and 2 studies used a single strain in the intervention (44, 54). The administration of the probiotic/synbiotic was done pre-operatively in 6 studies (35, 38, 42, 43, 49, 52), pre- and post-operatively in 8 studies (34, 36, 37, 44, 45, 48, 54, 56) and 3 studies focused on the post-operative period (46, 47, 57). Considering control groups, 4 studies used standard care (43–45, 57) while the remaining 13 used placebo (34–38, 42, 46–49, 52, 54, 56).

**Table 3:** Details of the included studies that assessed the impact on surgery outcomes.

Reference	Intervention Group			Control Group				Cancer Stage	Intervention		Outcome
	N°	Mean age (min-máx)	Male/ Female	Type	N°	Mean age (min-máx)	Male/ Female		Type	Dose/ Duration	
Horvat, M. 2010 (42)	48	62 (29-86)	19/29	Placebo	20	65 (52-78)	11/9	–	Synbiotic group = mixture of four *lactobacilli (Pediacoccus pentosaceus Leuconostoc mesenteroides Lactobacillus paracasei Lactobacillus plantarum)* + betaglucan, inulin, pectin and resistant starch Prebiotic group = betaglucan, inulin, pectin and resistant starch	40 billion *lactobacilli* plus 10 g plant fibers, twice a day for three days before the scheduled operation date.	Preoperative prebiotic administration had the same protective effect in preventing the postoperative inflammatory response (leukocytes and differential counts (lymphocyte/granulocyte ratio), fibrinogen and C-reactive protein) as mechanical bowel cleaning. Further prospective studies are needed to verify the effects of synbiotics.
Liu, Z. 2011 (34)	50	65	28/22	Placebo	50	66	31/19	I-III	*Lactobacillus plantarum, Lactobacillus acidophilus and Bifidobacterium longum*	2 g/day, in a total daily dose of 2.6x1014 CFU, 6 days preoperatively and 10 days post-operatively	Probiotics reduced the infection risk (p<0.01), improved gut barrier function (p<0.01) and reduced post-operative infectious complications (p<0.05).
Mangell, P. 2012 (54)	32	74 (70-80)	16/16	Placebo	32	70 (64-79)	20/12	–	*Lactobacillus* *plantarum 299v*	10^9^ CFU per ml, 100ml 8 days preoperatively and 5 days post-operatively	Lactobacillus plantarum299v was detected in the intestine, but no inhibitory effect on enteric bacteria, bacterial translocation, or postoperative complications was found.
Zhang, J. 2012 (35)	30	68 (45-87)	10/20	Placebo	30	62 (46-82)	14/16	I-III	*Enterococcus faecalis, Lactobacillus acidophilus and Bifidobacterium longum*	3 oral bifid triple viable capsules, each of which contained 0.21 g (108cfu/g), 3 times a day for 3 days before surgery (days -5 to -3)	Probiotics reduced the postoperative occurrence of infectious complications (p<0.05). Moreover, the probiotics maintained microbial colonization resistance and restricted bacterial translocation from the intestine.
Liu, Z. 2013 (36)	75	66	38/37	Placebo	75	62	40/35	I-III	*Lactobacillus plantarum, Lactobacillus acidophilus and Bifidobacterium longum*	2 g/day, in a total daily dose of 2.6x10^14^ CFU, 6 days preoperatively and 10 days post-operatively	Probiotics group had significantly lower bacterial translocation (p=0.027) and intestinal permeability (p=0.001=. Probiotics also reduced the rate of postoperative septicemia (p=0.017) and were associated with reduced serum zonulin concentrations (p=0.001).
Sadahiro, S. 2014 (44)	100	67	49/51	Standard Care	95	66	51/44	I-III	*Bifidobacterium bifidum*	Three tablets orally after each meal three times a day for 7 days before the operation and from postoperative D-5 to D-15.	The preventive effect of oral administration of probiotics on postoperative infection could not be confirmed.
Kotzampassi, K. 2015 (46)	84	66	57/27	Placebo	80	66	58/22	–	*Lactobacillus plantarum, Lactobacillus acidophilus, Bifidobacterium lactis and Saccharomyces boulardii*	1 capsule twice a day for 14 days post-operatively.	Administration of probiotics significantly decreased the rate of all postoperative major complications (p=0.010). Major benefit was found in the reduction of the rate of postoperative pneumonia (p=0.029), of surgical site infections (p=0.020) and of anastomotic leakage (0.031). The time until hospital discharge was shortened as well (p<0.0001).
Komatsu, S. 2016 (45)	168	69 (29-92)	92/76	Standard Care	194	69 (30-89)	118/76	I-IV	*Lactobacillus casei and Bifidobacterium breve* + galactoligosaccharides	4x10^10^ CFU L.casei + 1x10^10^ B.breve + 2,5g galactoligosaccharide, orally administered daily for 7–11 days before surgery and reintroduced at 2–7 postoperative days.	The preventive effect of oral administration of probiotics on postoperative infection could not be confirmed.
Tan, C. 2016 (52)	20	64	11/9	Placebo	20	68	13/7	0-III	*Lactobacillus lactis, Lactobacillus acidophilus, Lactobacillus casei*, *Bifidobacterium infantis, Bifidobacterium bifidum and Bifidobacterium longum*	30 billion CFU administered orally twice daily (1 sachet in the morning and 1 in the evening) 7 days prior to surgery	The treatment group demonstrated significantly faster return of normal gut function (48 h earlier than the placebo group) (p=0.022) The duration of hospital stay in the treatment group was also reduced (p=0.012).
Krebs, B. 2016 (43)	38	63 (43-87)	24/14	Standard Care	16	67 (52-78)	9/7	–	Synbiotic group = mixture of four *lactobacilli (Pediacoccus pentosaceus Leuconostoc mesenteroides Lactobacillus paracasei Lactobacillus plantarum)* + betaglucan, inulin, pectin and resistant starch Prebiotic group = betaglucan, inulin, pectin and resistant starch	1 sachet twice a day, 3 days prior to surgery.	No statistical differences in systemic inflammatory response measured by upper factors and no differences in postoperative course and complications rate were found.
Theodoropoulos, G. 2016 (47)	38	67	20/18	Placebo	37	69	23/14	0-IV	Mixture of four *lactobacilli (Pediacoccus pentosaceus Leuconostoc mesenteroides Lactobacillus paracasei Lactobacillus plantarum)* + betaglucan, inulin, pectin and resistant starch	2 g in 250 mL of water once a day for 15 days post-operatively	Synbiotic group had a better global score in the Gastrointestinal Quality of Life Index (p=0.01). The scores on the domain “diarrhea” were better in the synbiotic group after 3 (p=0.04) and 6 months (p=0.003). No significant effect was observed in the “constipation” domain.
Yang, Y. 2016 (37)	30	64	15/15	Placebo	30	62	12/18	0-III	*Enterococcus faecalis, Lactobacillus acidophilus and Bifidobacterium longum*	1x10^7^ CFU of each strain once a day for 12 days (5 prior to surgery and 7 post-operatively.	The days to first flatus (p=0.03) and the days to first defecation (p=0.03) were significantly improved in the probiotic treated patients. The incidence of diarrhea was significantly lower in probiotics group (p=0.04). There were no statistical differences in other infectious and non-infectious complication rates.
Flesch, A. 2017 (48)	49	65	18/31	Placebo	41	61	19/23	I-IV	*Lactobacillus rhamnosus, Lactobacillus acidophilus, Lactobacillus paracasei and Bifidobacterium lactis* + fructoligosaccharides	2 sachets twice a day for 5 days prior to surgery and 14 days post-operatively	The perioperative administration of synbiotics significantly reduced postoperative infection rates (p=0.002). The incidence of noninfectious postoperative complications wasn’t different between the study groups. There were no significant differences between the groups regarding mortality rates and re-hospitalization.
Polakowski, C. 2019 (49)	36	61	20/16	Placebo	37	59	19/18	I-III	*Lactobacillus rhamnosus, Lactobacillus acidophilus, Lactobacillus casei and Bifidobacterium lactis +* fructoligosaccharides	1 sachet twice a day for 7 days prior to the surgery	There were significant reductions in IL-6 and CRP levels in the synbiotic group (p<0.001). Postoperative infectious complications were reduced in the synbiotic group (p=0.02). Administration of synbiotics was also associated with reduced length of hospital stay (p<0.001) and use of antibiotics (p<0.001).
Xu, Q. 2019 (38)	30	61	20/10	Placebo	30	62	18/12	–	Bfiidus-Triple Viable Preparation (Inner Mongolia Shuangqi Pharmaceutical Co., Ltd., Inner Mongolia, China)	Once a day for 7 consecutive days prior to surgery.	Probiotics group had significantly lower bacterial translocation and intestinal permeability (p<0.05). The duration of post-operative fever, average heart rate at 7 days after surgery and first exhaust time were shorter and lower (p<0.05).
Bajramagic, S. 2019 (57)	39	–	–	Standard Care	38	–	–	III	*Lactobacillus plantarum, Lactobacillus acidophilus*, *Lactobacillus rhamnosus, Lactobacillus casei*, *Bifidobacterium lactis, Bifidobacterium breve, Bifidobacterium bifidum, and Streptococcus thermopilus*	2x1 capsules from the third postoperative day during the next thirty days, and then 1x1 for two weeks each next month to a total of one year.	There was a significant difference in the duration of postoperative hospitalization in the group of patients treated with probiotic (p<0.05). All complications were more present in the group of patients untreated with probiotic, with statistical significance shown only in the case of ileus (p<0.05).
Park, I. 2020 (56)	29	60	19/10	Placebo	31	61	13/18	I-IV	*Lactobacillus plantarum, Lactobacillus casei and Bifidobacterium animalis*	Twice a day for 1 week prior to surgery and 21 days post-operatively.	The probiotic group’s “Anterior Resection Syndrome” Score showed an improving trend (p=0.063) particularly for flatus control (p=0.03). Serum zonulin levels significantly decreased with probiotics (p=0.035).

##### 3.4.1.1 Post-operative complications

Fourteen studies (34–37, 43–46, 48, 49, 52, 54, 56, 57) assessed the incidence of post-operative infectious complications such as wound infection, septicemia, and pneumonia. Among them, 9 studies (34–37, 46, 48, 49, 56, 57) reported that the supplementation of probiotics or synbiotics could decrease the incidence of post-operative infectious complications, with significant results being observed in all but 2 of the studies (37, 57). Additionally, 5 studies (43–45, 52, 54) found no differences between the intervention and the control group.

In a study by Flesch et al., where the intervention group took synbiotics for 5 days before surgical procedure and for 14 days after surgery, it was observed that only one patient in the synbiotics group presented surgical wound infection, while 9 such cases were diagnosed in the control group (*p*=0.002). Furthermore, there was a significant difference between groups in relation to other infectious complications such as intra-abdominal abscess (n=3) and pneumonia (n=4) in the control group and no cases in the synbiotics group (*p*=0.001) (48).

A study by Liu et al., where patients received a mixture of probiotics for 6 days preoperatively and 10 days post-operatively, also reported a significant difference in postoperative infectious complications between the intervention and the control group, being the incidence of post-operative septicemia 73% in the control group and 55% in the probiotics group (*p*= 0.017) (36). In contrast, Komatsu et al. reported no statistical differences in postoperative infectious complications between the intervention and control group where synbiotics were administered from day 7 to day 11 before surgery and reintroduced from day 2 to day 7 postoperative (45). Mangell et al. also noted a higher number of complications in the placebo compared with the intervention group, where an administration of a single strain for 8 days preoperatively and 5 days postoperative was performed, although, this difference did not reach statistical significance (54). Four studies (34, 36, 38, 56) demonstrated a lower bacterial translocation and intestinal permeability in the intervention group and 2 of the studies reported lower zonulin levels which is used as a biomarker of impaired gut function barrier (36, 56).

Regarding non-infectious postoperative complications such as diarrhea, ileus, and anatomic leakage, 8 studies (34, 36–38, 46, 49, 56, 57) indicated that the supplementation of probiotics and synbiotics could decrease their incidence, with all studies showing statistically significant results but one (49). Additionally, 1 study (48) found no statistical difference between the intervention and the control group when considering non-infectious complications.

A study by Yang et al. reported a lower incidence of diarrhea in the intervention group (26.7%) compared to the placebo (53.3%) (*p*=0.035), after administration of probiotics for 12 days (5 prior to surgery and 7 postoperatively). The results concerning anastomotic leakage and abdominal distension were essentially quite comparable between the 2 groups (37).

Similarly, Bajramagic et al. also reported a lower number of non-infectious complications in the intervention group after administration of probiotics for 30 days starting on day 3 postoperative, but this difference was only statistically significant for ileus development (57).

Another study, where the intervention group received a mixture of probiotics for 6 days preoperatively and 10 days post-operatively, also observed a significant lower incidence of non-infectious complications, compared with the placebo group: diarrhea (10% vs. 30%, *p*< 0.05), abdominal cramping (26% vs. 38%, *p*< 0.05) and distension (22% vs. 36%, *p*< 0.05), and a shorter duration of pyrexia (>38.5°C) (5.9 days vs. 7.2 days, *p*< 0.05) (34).

Polakowski et al. also evaluated the incidence of postoperative non-infectious complications after synbiotic supplementation, for 7 days prior to surgery. Even though the incidence was higher in the control group, it did not reach statistical significance (*p*=0.42) (49).

Conversely, in the study by Flesch et al. the incidence of non-infectious postoperative complications such as nausea, vomiting, abdominal distension, ileus, diarrhea or constipation was not different between the study groups (*p*=0.161) (48).

##### 3.4.1.2 Return to normal gut function

Nine studies (34, 37, 42, 43, 46, 47, 52, 54, 56) evaluated the time to return to normal gut function. Six studies (34, 37, 46, 47, 52, 56) found that the supplementation of probiotics or synbiotics could significantly improve the return to normal gut function, and other 3 studies (42, 43, 54) found some improvements in the intervention group, but the results did not reach statistical difference.

A study by Tan et al., where the intervention group received a mixture of probiotics for 7 days prior to surgery, demonstrated a significantly earlier return of normal gut function compared to the placebo group (108.5 h vs. 156.5 h respectively, *p*=0.022) (52).

Another study reported a significant improvement in the days to first flatus (3.63 ± 0.67 days in the placebo group versus 3.27 ± 0.58 days in the probiotics group, *p* = 0.0274) and the days to first defecation (4.53 ± 1.11 days in the placebo group versus 3.87 ± 1.17 days in the probiotics group, *p* = 0.0268), after probiotics administration for 12 days (5 prior to surgery and 7 postoperative) (37).

In a study by Liu et al. a shorter time to first defecation when comparing the supplementation of a mixture of probiotics for 6 days preoperatively and 10 days post-operatively to placebo (3.3 days vs. 4.2 days, *p*< 0.05) was reported (34).

Horvat et al. demonstrated that patients receiving synbiotics and prebiotics twice a day for 3 days prior to surgery, passed flatus and stool after the operation earlier than the control. However, this difference did not reach statistical difference (2.3 days with synbiotics, 2.2 with prebiotics, and 2.5 days in the control, *p*=0.41) (42).

##### 3.4.1.3 Hospital length of stay

Eleven studies (34–37, 42, 43, 46, 48, 49, 52, 57) assessed the length of hospital stay after surgery. While 7 studies (36, 42, 43, 46, 49, 52, 57) found that the supplementation of probiotics or synbiotics could decrease the duration of hospital stay [significantly except for 2 studies (42, 43)], another 4 studies (34, 35, 37, 48) found no statistical difference between the intervention and the control group.

One study, where patients received probiotics for 7 days prior to surgery, the length of hospital stay was shorter for the intervention group in comparison to the placebo group (6.5 vs. 13 days, *p*=0.012) (52). Another study, where synbiotic supplementation was also administered for 7 days prior to surgery, reported a shorter length of hospitalization in the synbiotic group compared with the placebo group [3.0 (3-5) days, vs. 4.0 (3-21) days (*p <*0.001)] (49).

In contrast the study by Zhang et al. did not find differences in length of stay after probiotics administration for 3 days prior to surgery (35) contrary to studies by Krebs et al. (43) and Flesch et al. (48) that reported a shorter length of stay in the intervention groups, although they did not reach statistical significance.

##### 3.4.1.4 Usage of antibiotics

In terms of use of antibiotics, 2 studies (36, 49) found that the supplementation of probiotics or synbiotics could decrease the duration of antibiotic usage, and another 2 studies (34, 37) found no statistical difference between the intervention and the control group. Specifically, the study by Liu et al. reported a shorter duration of antibiotic therapy in the probiotics group compared with the control (5.69 vs. 7.29 days, *p*=0.001) (36). In agreement to Liu et al., Polakowski et al. also reported a shorter duration of antibiotic usage in the synbiotic group compared to the placebo group (1.42 vs. 3.74, *p*<0.001) (49). Contrary to these results, studies by Liu et al. and Yang et al. did not find significant differences in length of antibiotic therapy between the intervention and control groups (34, 37).

#### 3.4.2 Chemotherapy

Seven studies (39–41, 50, 51, 53, 55) assessed the effect of probiotics or synbiotics supplementation in CRC patients undergoing chemotherapy treatment. Details from each study are shown in **Table 4**. One study used synbiotics for the intervention group (50), 3 used a mixture of probiotics strains (39, 51, 55), 2 studies used kefir and one study used a single strain in the intervention group (53). For the control group, 2 studies used standard of care (51, 53), while the remaining 5 used some type of placebo (39–41, 50, 55).

**Table 4:** Details of the included studies that assessed the impact on chemotherapy and radiotherapy outcomes.

Reference	Intervention Group			Control Group				Cancer Stage	Intervention		Outcome
	N°	Mean age (min-máx)	Male/ Female	Type	N°	Mean age (min-máx)	Male/ Female		Type	Dose/ Duration	
Österlund, P. 2007 (53)	98	61 (35-74)	51/47	Standard Care	52	57 (31-75)	25/27	II-IV	*Lactobacillus rhamnosus GG*	1-2x10^10^ per day, twice a day during the 24 weeks of adjuvant cancer CT*.	Participants had less grade 3 or 4 diarrhea (p=0.027), reported less abdominal discomfort (p=0.025), needed less hospital care (p=0.021) and had fewer CT dose reductions due to bowel toxicity (p=0.0008).
Topuz, E. 2008 (40)	17	51 (19-75)	12/5	Placebo	20	58 (34-72)	12/8	II-IV	Kefir	250 ml of kefir twice a day after meals on the first 5 days of each CT cycle.	Kefir consumption made no statistically significant effect on serum proinflammatory cytokine levels and on the incidence of mucositis development.
Can, G. 2009 (41)	17	–	12/5	Placebo	20	–	12/8	II-IV	Kefir	250 ml of kefir twice a day after meals on the first 5 days of each CT cycle.	Kefir does not prevent or decrease gastrointestinal complaints in patients undergoing CT for colorectal cancer. No difference was found between the two groups for quality of life.
Mego, M. 2015 (55)	23	62 (45-75)	14/9	Placebo	23	64 (42-81)	12/11	–	*Lactobacillus plantarum, Lactobacillus acidophilus, Lactobacillus rhamnosus, Lactobacillus casei*, *Lactobacillus brevis, Bifidobacterium infantis, Bifidobacterium breve, Bifidobacterium bifidum, Bifidobacterium longum and Streptococcus thermopilus*	10×10^9^ CFU per capsule. 3×1 capsule per day orally for 12 weeks.	Administration of probiotics reduced the incidence of enterocolitis (p=0.49), of diarrhea (p=0.24) and of severe diarrhea (grade 3 or 4) (p=0.11). Usage of antidiarrheal drugs was also reduced (p=0.45). There was no infection caused by probiotics recorded. Results did not reach statistical significance.
Farshi Radvar F. 2020 (50)	19	58	13/6	Placebo	19	63	12/7	II-III	*Lactobacillus rhamnosus, Lactobacillus acidophilus*, *Lactobacillus bulgaricus, Lactobacillus casei, Bifidobacterium longum, Bifidobacterium breve and Streptococcus thermopilus* + fructoligosaccharides	1x10^8^ CFU twice a day before meals for 6 weeks starting 1 week before treatment to the end of radiotherapy.	Synbiotic supplementation caused improvement in global health status (p=0.60), symptom scale scores (p=0.75) and scores of functional scales (p=0.57). Nausea and vomiting (p=0.16), insomnia (p=0.25) and diarrhea (p=0.20) decreased slightly in the synbiotic group but increased significantly in the placebo group.
Mohebian, F. 2023 (51)	19	–	13/6	Standard Care	25	–	18/7	–	*Lactobacillus rhamnosus, Lactobacillus acidophilus*, *Lactobacillus bulgaricus, Lactobacillus casei, Bifidobacterium longum*, *Bifidobacterium breve and Streptococcus thermopilus* + fructoligosaccharides	1 capsule (500mg) twice a day for 1 week.	The number of defecations in the yogurt group with probiotics and yogurt was significantly lower than the control group p<0.05). The severity of diarrhea in the group with probiotics decreased more rapidly (p<0.05). Stool consistency in the group with probiotics was significantly better than the control (p<0.05).
Huang, F. 2023 (39)	50	58	24/26	Placebo	50	62	29/21	I-III	*Enterococcus faecalis, Lactobacillus acidophilus, Bacillus cereus and Bifidobacterium infantis*	1×3 tablets (1 capsule 3 times per day) from the 3^rd^ post-operative day to the end of the 1^st^ CT cycle.	Results showed that probiotics administration could effectively reduce CT-induced gastrointestinal complications, particularly in diarrhea (p<0.01).

The impact of probiotics or synbiotics supplementation on the incidence of diarrhea was assessed in all studies but one (40). Significant improvement of this side-effect was reported in 3 of the following studies: (1) Österlund et al. reported a lower incidence of diarrhea grade 3 – 4 in patients who received *Lactobacillus rhamnosus* twice a day for the 24 weeks of adjuvant chemotherapy (22% vs 37%, *p*=0.027) (53), comparing to the placebo group; (2) Mohebian et al. noted a lower severity of diarrhea and improved stool consistency in patients who took a mixture of probiotics twice a day for one week (51); and (3) Huang et al. also demonstrated a lower incidence of diarrhea in patients who received probiotics from day 3 postoperative to the end of the first neoadjuvant chemotherapy cycle, in a total of 6 weeks (16% vs. 40%, *p*=0,008) (39). Mego et al. also observed a lower incidence of overall diarrhea and lower incidence of grade 3 – 4 diarrhea when probiotics were administered 3 times a day for 12 weeks, however, these results did not reach statistical difference when compared to the placebo (0% vs. 17.4% *p*=0.11 and 39.1% vs. 60.9% *p*=0.24 respectively) (55). In contrast, a study by Can et al., where 250ml of kefir was consumed during the first 5 days of each chemotherapy cycle, reported that this intervention did not prevent diarrhea but increased constipation (41).

Besides lower incidence of diarrhea, the study by Huang et al. also reported, when compared to placebo, lower incidences of abdominal pain (6% vs 24%, *p*=0.025), abdominal distension (10% vs 28%, p=0.041) and constipation (8% vs 28%, *p*=0.019) in the group who took probiotics (39). Furthermore, Österlund et al. also demonstrated a lower abdominal discomfort resulting from flatulence and less abdominal distension in patients who took *Lactobacillus rhamnosus* (2% vs. 12%, *p*=0,025). This study also showed statistical significance for less chemotherapy-dose reductions due to bowel toxicity (21% vs 47%, *p*=0,008) in the intervention group (53). Accordingly, Farshi Radvar et al. demonstrated that synbiotic administration for 6 weeks, starting one week before chemoradiotherapy, decreased the incidence of symptoms such as nausea, vomiting, appetite loss and diarrhea, even though these results weren’t statistically significant, the placebo group has significant increases in these symptoms (50).

One study assessed the impact of kefir supplementation on mucositis development and reported no preventive effect of supplementation during the first 5 days of each chemotherapy cycle (40).

#### 3.4.3 Radiotherapy

Farshi Radvar et al. assessed the impact of synbiotics supplementation during radiotherapy, in rectal cancer patients undergoing neoadjuvant chemoradiotherapy. All of the participants received pelvic radiotherapy 5 times a week for 5 to 6 weeks and an intravenous dose of chemotherapy daily for 5 days in the beginning and at the end of radiotherapy. Participants in the intervention group took synbiotics for 6 weeks starting 1 week before beginning chemoradiotherapy. Quality of life was assessed through the European Organization for Cancer Research and Treatment of Cancer’s 30-item quality of life questionnaire version 3.0 which is composed of 3 scales: global health status, functional scale, and symptom scale (50, 58).

The results showed that, in terms of global health status, the synbiotic group had a higher improvement (69.73 to 74.12; *p*=0.39) compared to the control group (68.42 to 68.85; *p*=0.96) but the results weren’t of statistical significance (*p*=0.60). No improvements were observed in the functional scale but the synbiotic group had a decrease in the overall mean of the symptom scale (18.45 to 16.95; *p*=0.56) while the control group had an increase (21.37 to 24.88; *p*=0.29), although the results showed no statistical significance between the 2 groups (*p*=0.22). Particularly, nausea and vomiting (4.38 to 3.50; *p*=0.71) and diarrhea (33.33 to 26.31; *p*=0.49) decreased slightly in the synbiotic group and increased [(10.52 to 17.54; *p*=0.17), and (45.51 to 57.89; *p*=0.27) respectively] in the placebo group. This study also evaluated the quality of life in both groups, showing that the synbiotic group had a bigger increase in this parameter compared to the placebo group, but no statistically significant difference was reached (69.73 to 74.12 vs. 68.42 to 68.85, *p*=0.60) (50).

## 4 Discussion

CRC treatment has evolved in recent years, yet the accompanying side-effects still significantly impact patients’ quality of life and prognosis (6, 59). For this reason, it remains imperative to explore solutions that may decrease the occurrence of associated toxicity and complications in order to achieve a more successful outcome (4). In the present systematic review, we aimed to critically examine the current scientific evidence on the impact of pre-, pro-, and synbiotics used for modulating the microbiota, in CRC patients undergoing treatment, and to determine the potential therapeutic use of such approach.

Gut microbiota, specifically dysbiosis, has been associated with the development and progression of CRC (60). Specific bacteria such as *Fusobacterium nucleatum, Escherichia coli, Bacteroides fragilis, Streptococcus gallolyticus* and *Peptostreptococcus anaerobius* are frequent in CRC patients and have been linked with its development in various studies. Studies show that dysbiosis and the presence of these bacteria may alter the inflammatory, genomic, and metabolic processes in the host in a way that promotes carcinogenesis through different mechanisms such as the ability to induce DNA damage, interference with the DNA damage repair, impact on signaling pathways and immune suppression (60–63). Dysbiosis has also been observed in cancer patients undergoing immunotherapy and chemotherapy and has been associated with the efficacy of these treatments and their gastrointestinal toxicity and side-effects (62, 64). Consequently, gut microbiota modulation, in order to restore gut microbiota balance, through pre-, pro- and synbiotics has been studied as a potential therapeutic agent, potentiating cancer treatment effect or preventing and managing treatment-related toxicity or complications (65, 66).

Surgery remains the primary treatment for nearly all CRC patients. Although effective, surgery can lead to postoperative infectious or non-infectious complications that may impact prognosis (67, 68). In this systematic review, studies showed that the probiotics and synbiotics supplementation can have a role in reducing the incidence of postoperative complications. However, some evidence remains contradictory, and no conclusions can be drawn regarding the optimal formulation, duration and dosage of the intervention. Studies with shorter intervention duration and those using only one strain of probiotics appeared to yield less significant results (43, 44, 54), suggesting that a mixture of probiotic strains for a longer period of time may be more effective to reduce the incidence of postoperative complications. Accordingly, the following three systematic reviews and meta-analyses reported that the supplementation of probiotics and synbiotics can reduce the incidence of postoperative complications in CRC patients: (1) Chen et al. reported that probiotic or synbiotic administration significantly reduced the risk of developing postoperative infectious complications by 37% (RR = 0.63; 95%CI: 0.54–0.74) (28)^;^ (2) Veziant et al. reported that there were significantly fewer infectious complications in the probiotic or synbiotic group (RR = 0.59; 95%CI: 0.47–0.75) (29)^;^ (3) Araújo et al. reported that probiotic supplementation reduced the incidence of surgical site infection (OR = 0.53; 95%CI: 0.36 - 0.78) (31). These systematic reviews and meta-analyses also highlight that more evidence is needed regarding which strains of probiotics to use and what is the ideal intervention duration.

Probiotics and synbiotics may also facilitate a faster return to normal gut function, reduce hospital length of stay and decrease antibiotics usage after surgery, as evidenced by the majority of studies (34, 36, 37, 46, 47, 49, 52, 56, 57). However, three studies showed no significant results and (42, 43, 48) other two showed no difference between the control and intervention groups (35, 37). The heterogeneity between studies, considering sample size and type, dosage and duration of the intervention may explain the differences between the results. A systematic review and meta-analysis by Amitay et al. reported that perioperative probiotics/synbiotics administration was associated with faster return to normal gut function, shorter postoperative antibiotics use, and shorter length of hospital stay (30). Zeng et al. also reported a shorter duration of antibiotic therapy but found no statistical differences in hospital length of stay (26) whereas An et al. concluded that probiotics may result in little to no difference in hospital length of stay after colorectal cancer surgery (69).

Both chemotherapy and radiotherapy can lead to several toxicity-related side-effects. Their dose or intensity reductions may thus be necessary, which in turn can result in less efficient outcome. In the past years, studies and systematic reviews have reported that gut modulation interventions can reduce the incidence of cancer treatment-related side-effects such as diarrhea and mucositis. However, most studies have included several types of cancers and not only CRC patients, which weakens conclusions, as results cannot be extrapolated (70–72).

This systematic review includes all studies that assessed the impact of probiotic or synbiotic supplementation in chemotherapy or radiotherapy exclusively in CRC patients. It was found that these interventions may have a potential role in alleviating gastrointestinal symptoms and overall quality of life of these patients (39, 50, 51, 53). Two studies using kefir in the intervention group, reported no improvements, as so kefir may not be an effective approach (40, 41). One study found no statistical significance when comparing probiotics to placebo, but this study ended prematurely due to slow accrual with only 46 out of the planned 220 patients, which may have compromised statistical power (55). Mahdavi et al. (27) reported that probiotics were not related to diarrhea incidence in patients undergoing chemotherapy, but, this systematic review only included three studies, compared to six studies included in the present review, which may explain the difference between the findings. Even though the evidence is promising, more studies with lower heterogeneity and exclusive for CRC patients are necessary to allow strong conclusions about the impact of these interventions in chemotherapy and radiotherapy toxicity and side-effects.

As previously mentioned, studies included in this review exhibit high heterogeneity between them in terms of used strains, dose, and duration of the intervention. Nonetheless, it can be observed that among studies where probiotics or synbiotics supplementation had a beneficial effect, some strains were present in the intervention across most of them. This is the case of species such as *Lactobacillus rhamnosus, Lactobacillus acidophilus, Lactobacillus plantarum and Bifidobacterium lactis.* These are well known beneficial bacteria that help maintain a functional and structured gut barrier with preclinical studies showing that *Lactobacillus* spp. and *Bifidobacterium* spp. have anticancer functions such as inhibition of cell proliferation, induction of cancer cell apoptosis, modulation of host immunity and reduction of inflammation (62, 66). Furthermore, these strains are butyrate-producing bacteria which can repair and enhance gut barrier function and appears to inhibit proliferation of CRC cells (61). Taking this into account, administration of a combination of *Lactobacillus rhamnosus, Lactobacillus acidophilus, Lactobacillus plantarum, and Bifidobacterium lactis* can be recommended for these types on interventions.

In terms of efficacy of probiotics versus synbiotics in CRC treatment, more robust evidence is needed in order to make stronger conclusions. The prebiotics contained in the synbiotics used in the studies included in the present review varied from fructoligosaccharides and galactoligosaccharides to betaglucan, inulin, pectin, and resistant starch with fructoligosaccharides being the one present in most of the studies with results of statistical significance that used synbiotics. The consumption of these types of prebiotics has been associated with increased counts of *Lactobacillus* spp. and *Bifidobacterium* spp. and as a result, higher levels of short-chain fatty acids including butyrate which, as previously mentioned, have anticancer properties (73, 74). Yet, in the present review, interventions with synbiotics do not appear to be more efficient than interventions with probiotics. Again, randomized controlled studies with less heterogeneity and larger sample sizes are needed in order to determine which gut modulation intervention is more adequate to improve CRC treatment.

Some studies have showed that gut microbiota composition differs across CRC progression. Notably patients with early-stage CRC (stage I-II) exhibit a distinct microbiota compared to those with late-stage CRC (stage III-IV) (9, 75). This raises questions about whether the efficacy of pre-, pro-, and synbiotic supplementation differs based on CRC stage. The majority of the studies encompassed in this systematic review included patients with different stages of CRC but didn’t divide them accordingly. A systematic review from Dikeocha et al. (24) reported that probiotics supplementation has beneficial effects regardless of CRC stage. Nonetheless, future studies should take this information into consideration and compare the effectiveness of these types of interventions in different stages of CRC. Similarly, it has been noted that gut microbiota composition varies depending on the location of the CRC tumor with tumors on the left-side of the colon presenting a different microbiota than those on the right-side (76, 77). To our knowledge, no study accounted for this distinction by dividing the intervention group based on the tumor location. However, it would be interesting to future studies to investigate whether these factors influence the effectiveness of the intervention.

Although this systematic review also aimed to study the impact of microbiota modulation in immunotherapy, no studies meeting our inclusion criteria, specifically focusing on CRC patients undergoing immunotherapy were found. This treatment is a relatively new approach for CRC patients so it’s possible that research may still be ongoing, and results will be published upcoming years regarding the role of pre-, pro-, and synbiotic supplementation in CRC patients undergoing immunotherapy (7, 78). Nevertheless, a recent meta-analysis, that included 6 studies, reported that probiotics improved the efficacy of immune checkpoint inhibitors in non-small cell lung cancer patients with the intervention group having better overall survival and higher objective response rate and disease control rate (79).

One point that also has to be considered is the safety of these interventions in CRC patients. Prebiotics, probiotics and synbiotics are in general considered safe, specially the most common studied and used strains such as *Lactobacillus* and *Bifidobacterium* (14, 80). In addition, none of the studies included in this review reported major adverse reactions caused by the intervention. Yet, microbiota modulation may impact the prognosis, the immune function and toxicity, as so safe strains have to be confirmed and immunocompromised patients should be carefully monitored. More studies to assess the safety of gut modulation in this population are needed (81).

This systematic review has limitations, including high heterogeneity between intervention groups (strains, dose, and duration) and different primary outcomes, which compromise the accuracy of comparisons between studies. Most studies had a small sample size, and some had a short intervention time which decreases the probability of obtaining statistically significant results. Additionally, not all existing databases were searched, and only studies in English or Portuguese were full text screened and included in this review which may have led to a loss of relevant data.

Nonetheless, this review has its strengths. A comprehensive search was performed in several electronic databases and all current available evidence, including more recent studies, was analyzed. The search was not limited in terms of interval of years of publication, and it studied the impact of pre-, pro-, and synbiotic supplementation in different types of CRC treatment and not just one specific treatment.

## 5 Conclusion

In conclusion, the comprehensive analysis conducted in this systematic review suggests that supplementation with prebiotics, probiotics, and synbiotics may be beneficial for patients undergoing treatment for CRC. There is moderate evidence that this type of intervention in CRC patients may potentially facilitate return to normal gut function and decrease the occurrence of both infectious and non-infectious postoperative complications, reduce hospital length of stay, and mitigate antibiotic usage. Furthermore, there is also some evidence suggesting that probiotic and synbiotic administration may help lessen some side effects, mainly diarrhea, associated with chemo- and radiotherapy. Interventions with more than one strain type, and longer duration, appear to be more effective. Randomized controlled studies with less heterogeneity and larger sample sizes are needed in order to determine the best approach regarding strain selection, dosage, and duration of the intervention in gut modulation interventions in CRC patients.

## Data availability statement

The original contributions presented in the study are included in the article/**Supplementary Material**. Further inquiries can be directed to the corresponding author.

## Author contributions

MM: Conceptualization, Data curation, Investigation, Methodology, Writing – original draft, Writing – review & editing. MC: Data curation, Investigation, Writing – review & editing. MC: Conceptualization, Investigation, Methodology, Supervision, Writing – review & editing. NP: Writing – review & editing. TS: Writing – review & editing. SG: Writing – review & editing. AM: Writing – review & editing. PR: Formal analysis, Supervision, Validation, Writing – review & editing.
