# Supplementary material for: The impact of pre-, pro- and synbiotics supplementation in colorectal cancer treatment: a systematic review
Source: Front Oncol. 2024 May 14;14:1395966. doi: 10.3389/fonc.2024.1395966 (PMC11130488; doi:10.3389/fonc.2024.1395966)
Supplement: Supplementary file 1 [file DataSheet_1.docx]

| Search Strategy  **Appendix 1:** Search strategy for the different databases | |
| --- | --- |
| ****Pubmed**** | **(Colonic Neoplasms[MeSH Terms]) OR (Colorectal Neoplasms[MeSH Terms])) OR (Rectal Neoplasms[MeSH Terms])) OR (Anus Neoplasms[MeSH Terms])) OR (Colorectal Neoplasms, Hereditary Nonpolyposis[MeSH Terms])) OR (Sigmoid Neoplasms[MeSH Terms])) OR (Colitis-Associated Neoplasms[MeSH Terms])) OR (Colonic Neoplasm*[Title/Abstract])) OR (Colon Neoplas*[Title/Abstract])) OR (Cancer of Colon[Title/Abstract])) OR (Colon Cancer*[Title/Abstract])) OR (Colonic Cancer*[Title/Abstract])) OR (Colon Adenocarcinoma*[Title/Abstract])) OR (Colorectal Neoplasm*[Title/Abstract])) OR (Colorectal Tumor*[Title/Abstract])) OR (Colorectal Cancer*[Title/Abstract])) OR (Colorectal Carcinoma*[Title/Abstract])) OR (Rectal Neoplasm*[Title/Abstract])) OR (Rectum Neoplasm*[Title/Abstract])) OR (Rectal Tumor*[Title/Abstract])) OR (Cancer of Rectum[Title/Abstract])) OR (Rectum Cancer*[Title/Abstract])) OR (Rectal Cancer*[Title/Abstract])) OR (Anal Neoplasm*[Title/Abstract])) OR (Anus Neoplasm*[Title/Abstract])) OR (Anal Cancer*[Title/Abstract])) OR (Cancer of Anus[Title/Abstract])) OR (Anus Cancer*[Title/Abstract])) OR (Familial Nonpolyposis Colon Cancer[Title/Abstract])) OR (Hereditary Nonpolyposis Colorectal Neoplasms[Title/Abstract])) OR (Lynch Syndrome[Title/Abstract])) OR (Hereditary Nonpolyposis Colorectal Cancer[Title/Abstract])) OR (Hereditary Nonpolyposis Colon Cancer[Title/Abstract])) OR (Sigmoid Neoplasm*[Title/Abstract])) OR (Sigmoid Colon Neoplasm*[Title/Abstract])) OR (Sigmoid Cancer[Title/Abstract])) OR (Sigmoidal Cancer[Title/Abstract])) OR (Sigmoid Colon Cancer[Title/Abstract])) OR (Cancer of Sigmoid[Title/Abstract])) OR (Colitis Associated Neoplasm*[Title/Abstract])) OR (Colitis-Associated Neoplasm*[Title/Abstract])) OR (Colitis-Associated Colorectal Cancer*[Title/Abstract])) OR (Colitis Associated Colorectal Cancer*[Title/Abstract])) OR (Colitis-Associated Cancer*[Title/Abstract])) OR (Colitis Associated Cancer*[Title/Abstract])) OR (Colitis-Associated Colon Cancer*[Title/Abstract])) OR (Colitis Associated Colon Cancer*[Title/Abstract])) AND (Prebiotics[MeSH Terms]) OR (Probiotics[MeSH Terms])) OR (Synbiotics[MeSH Terms])) OR (Lactobacillus[MeSH Terms])) OR (Bifidobacterium[MeSH Terms])) OR (Prebiotic[Title/Abstract])) OR (Probiotics[Title/Abstract])) OR (Synbiotics[Title/Abstract])) OR (Lactobacillus[Title/Abstract])) OR (Bifidobacterium[Title/Abstract])) OR (symbiotic[Title/Abstract]))) AND (Radiotherapy[MeSH Terms]) OR (Immunotherapy[MeSH Terms])) OR (Immune Checkpoint Inhibitors[MeSH Terms])) OR (Antineoplastic Agents[MeSH Terms])) OR (Colorectal Surgery[MeSH Terms])) OR (Postoperative Complications[MeSH Terms])) OR (Surgical Wound Infection[MeSH Terms])) OR (Diarrhea[MeSH Terms])) OR (Nausea[MeSH Terms])) OR (Postoperative Nausea and Vomiting[MeSH Terms])) OR (Vomiting[MeSH Terms])) OR (Signs and Symptoms, Digestive[MeSH Terms])) OR (Mucositis[MeSH Terms])) OR (Quality of Life[MeSH Terms])) OR (Biomarkers[MeSH Terms])) OR (Biomarkers, Tumor[MeSH Terms])) OR (Radiotherapy*[Title/Abstract])) OR (Radiation Therapy*[Title/Abstract])) OR (Radiation Treatment*[Title/Abstract])) OR (Targeted Radiotherapy*[Title/Abstract])) OR (Targeted Radiation Therapy*[Title/Abstract])) OR (Immunotherapy*[Title/Abstract])) OR (Immune Checkpoint Inhibitor*[Title/Abstract])) OR (Immune Checkpoint Blockers[Title/Abstract])) OR (Immune Checkpoint Inhibition[Title/Abstract])) OR (CTLA-4 Inhibitor*[Title/Abstract])) OR (PD-L1 Inhibitor*[Title/Abstract])) OR (Antineoplastic Agent*[Title/Abstract])) OR (Anticancer Agent*[Title/Abstract])) OR (Antineoplastic Drug*[Title/Abstract])) OR (Antitumor Drug*[Title/Abstract])) OR (Cancer Chemotherapy Agent*[Title/Abstract])) OR (Cancer Chemotherapy Drug*[Title/Abstract])) OR (Chemotherapeutic Anticancer Agent*[Title/Abstract])) OR (Chemotherapeutic Anticancer Drug[Title/Abstract])) OR (Cancer Chemotherapy Drug[Title/Abstract])) OR (Antitumor Agent[Title/Abstract])) OR (Colorectal Surgery[Title/Abstract])) OR (Colon[Title/Abstract] AND Rectal Surgery Specialty[Title/Abstract])) OR (Colon Surgery Specialty[Title/Abstract])) OR (Rectal Surgery Specialty[Title/Abstract])) OR (Postoperative Complication*[Title/Abstract])) OR (Surgical Wound Infection*[Title/Abstract])) OR (Surgical Site Infection*[Title/Abstract])) OR (Postoperative Wound Infection*[Title/Abstract])) OR (Diarrhea*[Title/Abstract])) OR (Nausea[Title/Abstract])) OR (Postoperative Nausea[Title/Abstract] AND Vomiting[Title/Abstract])) OR (Postoperative Emesis[Title/Abstract])) OR (Postoperative Vomiting[Title/Abstract])) OR (Postoperative Nausea[Title/Abstract])) OR (Vomiting[Title/Abstract])) OR (Emesis[Title/Abstract])) OR (Signs[Title/Abstract] AND Symptoms, Digestive[Title/Abstract])) OR (Mucositis[Title/Abstract])) OR (Mucositides[Title/Abstract])) OR (Life Quality[Title/Abstract])) OR (Health-Related Quality Of Life[Title/Abstract])) OR (Health Related Quality Of Life[Title/Abstract])) OR (HRQOL[Title/Abstract])) OR (Quality of Life[Title/Abstract])) OR (Biomarker*[Title/Abstract])) OR (Biological Marker*[Title/Abstract])) OR (Biologic Marker*[Title/Abstract])) OR (Immunologic Marker*[Title/Abstract])) OR (Immune Marker*[Title/Abstract])) OR (Tumor Biomarker*[Title/Abstract])) OR (Carcinogen Marker*[Title/Abstract])) OR (Biological Tumor Marker*[Title/Abstract])) OR (Neoplasm Metabolite Marker*[Title/Abstract])) OR (Tumor Metabolite Marker*[Title/Abstract])) OR (Biologic Tumor Marker*[Title/Abstract])) OR (Cancer Biomarker*[Title/Abstract]))** |

| Search Strategy | |
| --- | --- |
| ****Scopus**** | **((TITLE-ABS-KEY(Prebiotic)) OR (TITLE-ABS-KEY(Probiotics)) OR (TITLE-ABS-KEY(Synbiotics)) OR (TITLE-ABS-KEY(Lactobacillus)) OR (TITLE-ABS-KEY(Bifidobacterium))) AND (((TITLE-ABS-KEY("Radiotherapy*")) OR (TITLE-ABS-KEY("Radiation Therapy*")) OR (TITLE-ABS-KEY("Radiation Treatment*")) OR (TITLE-ABS-KEY("Targeted Radiotherapy*")) OR (TITLE-ABS-KEY("Targeted Radiation Therapy*")) OR (TITLE-ABS-KEY("Immunotherapy*")) OR (TITLE-ABS-KEY("Immune Checkpoint Inhibitor*")) OR (TITLE-ABS-KEY("Immune Checkpoint Blockers"))) OR (((TITLE-ABS-KEY("Cancer Biomarker*")) OR (TITLE-ABS-KEY("Biologic Tumor Marker*")) OR (TITLE-ABS-KEY("Tumor Metabolite Marker*")) OR (TITLE-ABS-KEY("Neoplasm Metabolite Marker*")) OR (TITLE-ABS-KEY("Biological Tumor Marker*")) OR (TITLE-ABS-KEY("Carcinogen Marker*")) OR (TITLE-ABS-KEY("Tumor Biomarker*")) OR (TITLE-ABS-KEY("Immune Marker*")) OR (TITLE-ABS-KEY("Immunologic Marker*")) OR (TITLE-ABS-KEY("Biologic Marker*")) OR (TITLE-ABS-KEY("Biological Marker*")) OR (TITLE-ABS-KEY("Biomarker*")) OR (TITLE-ABS-KEY("Quality of Life")) OR (TITLE-ABS-KEY("HRQOL")) OR (TITLE-ABS-KEY("Health Related Quality Of Life")) OR (TITLE-ABS-KEY("Health-Related Quality Of Life")) OR (TITLE-ABS-KEY("Life Quality")) OR (TITLE-ABS-KEY("Mucositides")) OR (TITLE-ABS-KEY("Mucositis")) OR (TITLE-ABS-KEY("Signs and Symptoms, Digestive")) OR (TITLE-ABS-KEY("Emesis"))) OR (TITLE-ABS-KEY("Vomiting")) OR (TITLE-ABS-KEY("Postoperative Nausea")) OR (TITLE-ABS-KEY("Postoperative Vomiting")) OR (TITLE-ABS-KEY("Postoperative Emesis")) OR (TITLE-ABS-KEY("Postoperative Nausea and Vomiting")) OR (TITLE-ABS-KEY("Nausea")) OR (TITLE-ABS-KEY("Diarrhea*")) OR (TITLE-ABS-KEY("Postoperative Wound Infection*")) OR (TITLE-ABS-KEY("Surgical Site Infection*")) OR (TITLE-ABS-KEY("Surgical Wound Infection*")) OR (TITLE-ABS-KEY("Postoperative Complication*")) OR (TITLE-ABS-KEY("Rectal Surgery Specialty")) OR (TITLE-ABS-KEY("Colon Surgery Specialty")) OR (TITLE-ABS-KEY("Colon and Rectal Surgery Specialty")) OR (TITLE-ABS-KEY("Colorectal Surgery")) OR (TITLE-ABS-KEY("Antitumor Agent")) OR (TITLE-ABS-KEY("Cancer Chemotherapy Drug")) OR (TITLE-ABS-KEY("Chemotherapeutic Anticancer Drug")) OR (TITLE-ABS-KEY("Immune Checkpoint Inhibition")) OR (TITLE-ABS-KEY("CTLA-4 Inhibitor*")) OR (TITLE-ABS-KEY("PD-L1 Inhibitor*")) OR (TITLE-ABS-KEY("Antineoplastic Agent*")) OR (TITLE-ABS-KEY("Anticancer Agent*")) OR (TITLE-ABS-KEY("Antineoplastic Drug*")) OR (TITLE-ABS-KEY("Antitumor Drug*")) OR (TITLE-ABS-KEY("Cancer Chemotherapy Agent*")) OR (TITLE-ABS-KEY("Cancer Chemotherapy Drug*")) OR (TITLE-ABS-KEY("Chemotherapeutic Anticancer Agent*")))) AND (((TITLE-ABS-KEY("Familial Nonpolyposis Colon Cancer")) OR (TITLE-ABS-KEY("Hereditary Nonpolyposis Colorectal Neoplasms")) OR (TITLE-ABS-KEY("Lynch Syndrome")) OR (TITLE-ABS-KEY("Hereditary Nonpolyposis Colorectal Cancer")) OR (TITLE-ABS-KEY("Hereditary Nonpolyposis Colon Cancer")) OR (TITLE-ABS-KEY("Sigmoid Neoplasm*")) OR (TITLE-ABS-KEY("Sigmoid Colon Neoplasm*")) OR (TITLE-ABS-KEY("Sigmoid Cancer")) OR (TITLE-ABS-KEY("Sigmoidal Cancer")) OR (TITLE-ABS-KEY("Sigmoid Colon Cancer")) OR (TITLE-ABS-KEY("Cancer of Sigmoid")) OR (TITLE-ABS-KEY("Colitis Associated Neoplasm*")) OR (TITLE-ABS-KEY("Colitis-Associated Neoplasm*")) OR (TITLE-ABS-KEY("Colitis-Associated Colorectal Cancer*")) OR (TITLE-ABS-KEY("Colitis Associated Colorectal Cancer*")) OR (TITLE-ABS-KEY("Colitis-Associated Cancer*")) OR (TITLE-ABS-KEY("Colitis Associated Cancer*")) OR (TITLE-ABS-KEY("Colitis-Associated Colon Cancer*")) OR (TITLE-ABS-KEY("Colitis Associated Colon Cancer*"))) OR ((TITLE-ABS-KEY("Colonic Neoplasm*")) OR (TITLE-ABS-KEY("Colon Neoplasm*")) OR (TITLE-ABS-KEY("Cancer of Colon")) OR (TITLE-ABS-KEY("Colon Cancer*")) OR (TITLE-ABS-KEY("Colonic Cancer*")) OR (TITLE-ABS-KEY("Colon Adenocarcinoma*")) OR (TITLE-ABS-KEY("Colorectal Neoplasm*")) OR (TITLE-ABS-KEY("Colorectal Tumor*")) OR (TITLE-ABS-KEY("Colorectal Cancer*")) OR (TITLE-ABS-KEY("Colorectal Carcinoma*")) OR (TITLE-ABS-KEY("Rectal Neoplasm*")) OR (TITLE-ABS-KEY("Rectum Neoplasm*")) OR (TITLE-ABS-KEY("Rectal Tumor*")) OR (TITLE-ABS-KEY("Cancer of Rectum")) OR (TITLE-ABS-KEY("Rectum Cancer*")) OR (TITLE-ABS-KEY("Rectal Cancer*")) OR (TITLE-ABS-KEY("Anal Neoplasm*")) OR (TITLE-ABS-KEY("Anus Neoplasm*")) OR (TITLE-ABS-KEY("Anal Cancer*")) OR (TITLE-ABS-KEY("Cancer of Anus")) OR (TITLE-ABS-KEY("Anus Cancer*"))))** |

| Search Strategy | |
| --- | --- |
| ****Cochrane**** | **#1 MeSH descriptor: [Colonic Neoplasms]**  **#2 MeSH descriptor: [Colorectal Neoplasms]**  **#3 MeSH descriptor: [Rectal Neoplasms]**  **#4 MeSH descriptor: [Anus Neoplasms]**  **#5 MeSH descriptor: [Colorectal Neoplasms, Hereditary Nonpolyposis]**  **#6 MeSH descriptor: [Sigmoid Neoplasms]**  **#7 MeSH descriptor: [Colitis-Associated Neoplasms]**  **#8 (Colonic Neoplasm*):ti,ab,kw**  **#9 (Colon Neoplasm*):ti,ab,kw**  **#10 (Cancer of Colon):ti,ab,kw**  **#11 (Colon Cancer*):ti,ab,kw**  **#12 (Colonic Cancer*):ti,ab,kw**  **#13 (Colon Adenocarcinoma*):ti,ab,kw**  **#14 (Colorectal Neoplasm*):ti,ab,kw**  **#15 (Colorectal Tumor*):ti,ab,kw**  **#16 (Colorectal Cancer*):ti,ab,kw**  **#17 (Colorectal Carcinoma*):ti,ab,kw**  **#18 (Rectal Neoplasm*):ti,ab,kw**  **#19 (Rectum Neoplasm*):ti,ab,kw**  **#20 (Rectal Tumor*):ti,ab,kw**  **#21 (Cancer of Rectum):ti,ab,kw**  **#22 (Rectum Cancer*):ti,ab,kw**  **#23 (Rectal Cancer*):ti,ab,kw**  **#24 (Anal Neoplasm*):ti,ab,kw**  **#25 (Anus Neoplasm*):ti,ab,kw**  **#26 (Anal Cancer*):ti,ab,kw**  **#27 (Cancer of Anus):ti,ab,kw**  **#28 (Anus Cancer*):ti,ab,kw**  **#29 (Familial Nonpolyposis Colon Cancer):ti,ab,kw**  **#30 (Hereditary Nonpolyposis Colorectal Neoplasms):ti,ab,kw**  **#31 (Lynch Syndrome):ti,ab,kw**  **#32 (Hereditary Nonpolyposis Colorectal Cancer):ti,ab,kw**  **#33 (Hereditary Nonpolyposis Colon Cancer):ti,ab,kw**  **#34 (Sigmoid Neoplasm*):ti,ab,kw**  **#35 (Sigmoid Colon Neoplasm*):ti,ab,kw**  **#36 (Sigmoid Cancer):ti,ab,kw**  **#37 (Sigmoidal Cancer):ti,ab,kw**  **#38 (Sigmoid Colon Cancer):ti,ab,kw**  **#39 (Cancer of Sigmoid):ti,ab,kw**  **#40 (Colitis Associated Neoplasm*):ti,ab,kw**  **#41 (Colitis-Associated Neoplasm*):ti,ab,kw**  **#42 (Colitis-Associated Colorectal Cancer*):ti,ab,kw**  **#43 (Colitis Associated Colorectal Cancer*):ti,ab,kw**  **#44 (Colitis-Associated Cancer*):ti,ab,kw**  **#45 (Colitis Associated Cancer*):ti,ab,kw**  **#46 (Colitis-Associated Colon Cancer*):ti,ab,kw**  **#47 (Colitis Associated Colon Cancer*):ti,ab,kw**  **#48 #1 OR #2 OR #3 OR #5 OR #6 OR #7 OR #8 OR #9 OR #10 OR #11 OR #12 OR #13 OR #14 OR #15 OR #16 OR #17 OR #18 OR #19 OR #20 OR #21 OR #22 OR #23 OR #24 OR #25 OR #26 OR #27 OR #28 OR #29 OR #30 OR #31 OR #32 OR #33 OR #34 OR #35 OR #36 OR #37 OR #38 OR #39 OR #40 OR #41 OR #42 OR #43 OR #44 OR #45 OR #46 OR #47**  **#49 MeSH descriptor: [Prebiotics]**  **#50 MeSH descriptor: [Probiotics]**  **#51 MeSH descriptor: [Synbiotics]**  **#52 MeSH descriptor: [Lactobacillus]**  **#53 MeSH descriptor: [Bifidobacterium]**  **#54 (Prebiotic):ti,ab,kw**  **#55 (Probiotics):ti,ab,kw**  **#56 (Synbiotics):ti,ab,kw**  **#57 (Lactobacillus):ti,ab,kw**  **#58 (Bifidobacterium):ti,ab,kw**  **#59 #54 OR #55 OR #56 OR #57 OR #58**  **#60 MeSH descriptor: [Radiotherapy]**  **#61 MeSH descriptor: [Immunotherapy]**  **#62 MeSH descriptor: [Immune Checkpoint Inhibitors]**  **#63 MeSH descriptor: [Antineoplastic Agents]**  **#64 MeSH descriptor: [Colorectal Surgery]**  **#65 MeSH descriptor: [Postoperative Complications]**  **#66 MeSH descriptor: [Surgical Wound Infection]**  **#67 MeSH descriptor: [Diarrhea]**  **#68 MeSH descriptor: [Nausea]**  **#69 MeSH descriptor: [Postoperative Nausea and Vomiting]**  **#70 MeSH descriptor: [Vomiting]**  **#71 MeSH descriptor: [Signs and Symptoms, Digestive]**  **#72 MeSH descriptor: [Mucositis]**  **#73 MeSH descriptor: [Quality of Life]**  **#74 MeSH descriptor: [Biomarkers]**  **#75 MeSH descriptor: [Biomarkers, Tumor]**  **#76 (Radiotherapy*):ti,ab,kw**  **#77 (Radiation Therapy*):ti,ab,kw**  **#78 (Radiation Treatment*):ti,ab,kw**  **#79 (Targeted Radiotherapy*):ti,ab,kw**  **#80 (Targeted Radiation Therapy*):ti,ab,kw**  **#81 (Immunotherapy*):ti,ab,kw**  **#82 (Immune Checkpoint Inhibitor*):ti,ab,kw**  **#83 (Immune Checkpoint Blockers):ti,ab,kw**  **#84 (Immune Checkpoint Inhibition):ti,ab,kw**  **#85 (CTLA-4 Inhibitor*):ti,ab,kw**  **#86 (PD-L1 Inhibitor*):ti,ab,kw**  **#87 (Antineoplastic Agent*):ti,ab,kw**  **#88 (Anticancer Agent*):ti,ab,kw**  **#89 (Antineoplastic Drug*):ti,ab,kw**  **#90 (Antitumor Drug*):ti,ab,kw**  **#91 (Cancer Chemotherapy Agent*):ti,ab,kw**  **#92 (Cancer Chemotherapy Drug*):ti,ab,kw**  **#93 (Chemotherapeutic Anticancer Agent*):ti,ab,kw**  **#94 (Chemotherapeutic Anticancer Drug):ti,ab,kw**  **#95 (Cancer Chemotherapy Drug):ti,ab,kw**  **#96 (Antitumor Agent):ti,ab,kw**  **#97 (Colorectal Surgery):ti,ab,kw**  **#98 (Colon and Rectal Surgery Specialty):ti,ab,kw**  **#99 (Colon Surgery Specialty):ti,ab,kw**  **#100 (Rectal Surgery Specialty):ti,ab,kw**  **#101 (Postoperative Complication*):ti,ab,kw**  **#102 (Surgical Wound Infection*):ti,ab,kw**  **#103 (Surgical Site Infection*):ti,ab,kw**  **#104 (Postoperative Wound Infection*):ti,ab,kw**  **#105 (Diarrhea*):ti,ab,kw**  **#106 (Nausea):ti,ab,kw**  **#107 (Postoperative Nausea and Vomiting):ti,ab,kw**  **#108 (Postoperative Emesis):ti,ab,kw**  **#109 (Postoperative Vomiting):ti,ab,kw**  **#110 (Postoperative Nausea):ti,ab,kw**  **#111 (Vomiting):ti,ab,kw**  **#112 (Emesis):ti,ab,kw**  **#113 (Signs and Symptoms, Digestive):ti,ab,kw**  **#114 (Mucositis):ti,ab,kw**  **#115 (Mucositides):ti,ab,kw**  **#116 (Life Quality):ti,ab,kw**  **#117 (Health-Related Quality Of Life):ti,ab,kw**  **#118 (Health Related Quality Of Life):ti,ab,kw**  **#119 (HRQOL):ti,ab,kw**  **#120 (Quality of Life):ti,ab,kw**  **#121 (Biomarker*):ti,ab,kw**  **#122 (Biological Marker*):ti,ab,kw**  **#123 (Biologic Marker*):ti,ab,kw**  **#124 (Immunologic Marker*):ti,ab,kw**  **#125 (Immune Marker*):ti,ab,kw**  **#126 (Tumor Biomarker*):ti,ab,kw**  **#127 (Carcinogen Marker*):ti,ab,kw**  **#128 (Biological Tumor Marker*):ti,ab,kw**  **#129 (Neoplasm Metabolite Marker*):ti,ab,kw**  **#130 (Tumor Metabolite Marker*):ti,ab,kw**  **#131 (Biologic Tumor Marker*):ti,ab,kw**  **#132 (Cancer Biomarker*):ti,ab,kw**  **#133 #60 OR #61 OR #62 OR #63 OR #64 OR #65 OR #66 OR #67 OR #68 OR #69 OR #70 OR #71 OR #72 OR #73 OR #74 OR #75 OR #76 OR #77 OR #78 OR #79 OR #80 OR #81 OR #82 OR #83 OR #84 OR #85 OR #86 OR #87 OR #88 OR #89 OR #90 OR #91 OR #92 OR #93 OR #94 OR #95 OR #96 OR #97 OR #98 OR #99 OR #100 OR #101 OR #102 OR #103 OR #104 OR #105 OR #106 OR #107 OR #108 OR #109 OR #110 OR #111 OR #112 OR #113 OR #114 OR #115 OR #116 OR #117 OR #118 OR #119 OR #120 OR #121 OR #122 OR #123 OR #124 OR #125 OR #126 OR #127 OR #128 OR #129 OR #130 OR #131 OR #132**  **#134 #48 AND #59 AND #133** |

| Search Strategy | |
| --- | --- |
| ****Web of Science**** | **1: (TS=("Colonic Neoplasm*")) OR TS=("Colon Neoplasm*")) OR TS=("Cancer of Colon")) OR TS=("Colon Cancer*")) OR TS=("Colonic Cancer*")) OR TS=("Colon Adenocarcinoma*")) OR TS=("Colorectal Neoplasm*")) OR TS=("Colorectal Tumor*")) OR TS=("Colorectal Cancer*")) OR TS=("Colorectal Carcinoma*")) OR TS=("Rectal Neoplasm*")) OR TS=("Rectum Neoplasm*")) OR TS=("Rectal Tumor*")) OR TS=("Cancer of Rectum")) OR TS=("Rectum Cancer*")) OR TS=("Rectal Cancer*")) OR TS=("Anal Neoplasm*")) OR TS=("Anus Neoplasm*")) OR TS=("Anal Cancer*")) OR TS=("Cancer of Anus")) OR TS=("Anus Cancer*")) OR TS=("Familial Nonpolyposis Colon Cancer")) OR TS=("Hereditary Nonpolyposis Colorectal Neoplasms")) OR TS=("Lynch Syndrome")) OR TS=("Hereditary Nonpolyposis Colorectal Cancer")) OR TS=("Hereditary Nonpolyposis Colon Cancer")) OR TS=("Sigmoid Neoplasm*")) OR TS=("Sigmoid Colon Neoplasm*")) OR TS=("Sigmoid Cancer")) OR TS=("Sigmoidal Cancer")) OR TS=("Sigmoid Colon Cancer")) OR TS=("Cancer of Sigmoid")) OR TS=("Colitis Associated Neoplasm*")) OR TS=("Colitis-Associated Neoplasm*")) OR TS=("Colitis-Associated Colorectal Cancer*")) OR TS=("Colitis Associated Colorectal Cancer*")) OR TS=("Colitis-Associated Cancer*")) OR TS=("Colitis Associated Cancer*")) OR TS=("Colitis-Associated Colon Cancer*")) OR TS=("Colitis Associated Colon Cancer*"**  **2: (TS=(Prebiotic*)) OR TS=(Probiotic*)) OR TS=(Synbiotic*)) OR TS=(Lactobacillus)) OR TS=(Bifidobacterium)**  **3: (TS=(Radiotherapy*)) OR TS=("Radiation Therapy*")) OR TS=("Radiation Treatment*")) OR TS=("Targeted Radiotherapy*")) OR TS=("Targeted Radiation Therapy*")) OR TS=(Immunotherapy*)) OR TS=("Immune Checkpoint Inhibitor*")) OR TS=("Immune Checkpoint Blockers")) OR TS=("Immune Checkpoint Inhibition")) OR TS=("CTLA-4 Inhibitor*")) OR TS=("PD-L1 Inhibitor*")) OR TS=("Antineoplastic Agent*")) OR TS=("Anticancer Agent*")) OR TS=("Antineoplastic Drug*")) OR TS=("Antitumor Drug*")) OR TS=("Cancer Chemotherapy Agent*")) OR TS=("Cancer Chemotherapy Drug*")) OR TS=("Chemotherapeutic Anticancer Agent"*)) OR TS=("Chemotherapeutic Anticancer Drug")) OR TS=("Cancer Chemotherapy Drug")) OR TS=("Antitumor Agent")) OR TS=("Colorectal Surgery")) OR TS=("Colon and Rectal Surgery Specialty")) OR TS=("Colon Surgery Specialty")) OR TS=("Rectal Surgery Specialty")) OR TS=("Postoperative Complication*")) OR TS=("Surgical Wound Infection*")) OR TS=("Surgical Site Infection*")) OR TS=("Postoperative Wound Infection*")) OR TS=(Diarrhea*)) OR TS=(Nausea)) OR TS=("Postoperative Nausea and Vomiting")) OR TS=("Postoperative Emesis")) OR TS=("Postoperative Vomiting")) OR TS=("Postoperative Nausea")) OR TS=(Vomiting)) OR TS=(Emesis)) OR TS=(Signs and Symptoms, Digestive)) OR TS=(Mucositis)) OR TS=(Mucositides)) OR TS=("Life Quality")) OR TS=("Health-Related Quality Of Life")) OR TS=("Health Related Quality Of Life")) OR TS=(HRQOL)) OR TS=("Quality of Life")) OR TS=("Biomarker*")) OR TS=("Biological Marker*")) OR TS=("Biologic Marker*")) OR TS=("Immunologic Marker*")) OR TS=("Immune Marker*")) OR TS=("Tumor Biomarker*")) OR TS=("Carcinogen Marker*")) OR TS=("Biological Tumor Marker*")) OR TS=("Neoplasm Metabolite Marker*")) OR TS=("Tumor Metabolite Marker*")) OR TS=("Biologic Tumor Marker*")) OR TS=("Cancer Biomarker*")**  **4: #3 AND #2 AND #1** |

| Search Strategy | |
| --- | --- |
| ****CINAHL**** | **S1 MH Colonic Neoplasms OR MH Colorectal Neoplasms OR MH Rectal Neoplasms OR MH Anus Neoplasms OR MH Colorectal Neoplasms, Hereditary Nonpolyposis OR MH Sigmoid Neoplasms OR MH Colitis-Associated Neoplasms**  **S2 TI colonic neoplasms OR AB colonic neoplasms AND TI colon neoplasm OR AB colon neoplasm AND TI cancer of colon OR AB cancer of colon AND TI colon cancer OR AB colon cancer AND TI colonic cancer OR AB colonic cancer AND TI colon adenocarcinoma OR AB colon adenocarcinoma**  **S3 TI colorectal neoplasms OR AB colorectal neoplasms AND TI Colorectal Tumor OR AB Colorectal Tumor AND TI colorectal cancer OR AB colorectal cancer AND TI Colorectal Carcinoma OR AB colorectal carcinoma AND TI rectal neoplasms OR AB rectal neoplasms AND TI Rectum Neoplasm* OR AB Rectum Neoplasm***  **S4 TI rectal tumour OR AB rectal tumour AND TI Cancer of Rectum OR AB Cancer of Rectum AND TI rectum cancer OR AB rectum cancer AND TI rectal cancer OR AB rectal cancer AND TI anal neoplasms OR AB anal neoplasms AND TI Anus Neoplasm* OR AB Anus Neoplasm***  **S5 TI anal cancer OR AB anal cancer AND TI Cancer of Anus OR AB Cancer of Anus AND TI Anus Cancer OR AB Anus Cancer AND TI Familial Nonpolyposis Colon Cancer OR AB Familial Nonpolyposis Colon Cancer AND TI Hereditary Nonpolyposis Colorectal Neoplasms OR AB Hereditary Nonpolyposis Colorectal Neoplasms AND TI lynch syndrome OR AB lynch syndrome**  **S6 TI Hereditary Nonpolyposis Colorectal Cancer OR AB Hereditary Nonpolyposis Colorectal Cancer AND TI Hereditary Nonpolyposis Colon Cancer OR AB Hereditary Nonpolyposis Colon Cancer AND TI Sigmoid Neoplasm* OR AB Sigmoid Neoplasm* AND TI Sigmoid Colon Neoplasm* OR AB Sigmoid Colon Neoplasm* AND TI Sigmoid Cancer OR AB Sigmoid Cancer AND TI Sigmoidal Cancer OR AB Sigmoidal Cancer**  **S7 TI Sigmoid Colon Cancer OR AB Sigmoid Colon Cancer AND TI Cancer of Sigmoid OR AB Cancer of Sigmoid AND TI Colitis Associated Neoplasm* OR AB Colitis Associated Neoplasm* AND TI Colitis Associated Colorectal Cancer* OR AB Colitis Associated Colorectal Cancer* AND TI Colitis Associated Cancer* OR AB Colitis Associated Cancer* AND TI Colitis Associated Colon Cancer* OR AB Colitis Associated Colon Cancer***  **S8 S1 OR S2 OR S3 OR S4 OR S5 OR S6 OR S7**  **S9 MH Prebiotics OR MH Probiotics OR MH Synbiotics OR MH Lactobacillus OR MH Bifidobacterium**  **S10 TI prebiotics OR AB prebiotics AND TI probiotics OR AB Probiotics AND TI synbiotics OR AB Synbiotics AND TI lactobacillus OR AB Lactobacillus AND TI bifidobacterium OR AB bifidobacterium**  **S11 S9 OR S10**  **S12 MH Radiotherapy OR MH Immunotherapy OR MH Immune Checkpoint Inhibitors OR MH Antineoplastic Agents OR MH Colorectal Surgery OR MH Postoperative Complications OR MH Surgical Wound Infection OR MH Diarrhea OR MH Nausea OR MH ( Postoperative Nausea and Vomiting ) OR MH Vomiting**  **S13 MH ( Signs and Symptoms, Digestive ) OR MH Mucositis OR MH Quality of Life OR MH Biomarkers OR MH Biomarkers, Tumor**  **S14 TI Radiotherapy* OR AB Radiotherapy* AND TI Radiation Therapy* OR AB Radiation Therapy* AND TI Radiation Treatment* OR AB Radiation Treatment* AND TI Targeted Radiotherapy* OR AB Targeted Radiotherapy* AND TI Targeted Radiation Therapy* OR AB Targeted Radiation Therapy* AND TI Immunotherapy* OR AB Immunotherapy***  **S15 TI Immune Checkpoint Inhibitor* OR AB Immune Checkpoint Inhibitor* AND TI Immune Checkpoint Blockers OR AB Immune Checkpoint Blockers AND TI Immune Checkpoint Inhibition OR AB Immune Checkpoint Inhibition AND TI CTLA-4 Inhibitor* OR AB CTLA-4 Inhibitor* AND TI PD-L1 Inhibitor* OR AB PD-L1 Inhibitor* AND TI Antineoplastic Agent* OR AB Antineoplastic Agent***  **S16 TI Anticancer Agent* OR AB Anticancer Agent* AND TI Antineoplastic Drug* OR AB Antineoplastic Drug* AND TI Antitumor Drug* OR AB Antitumor Drug* AND TI Cancer Chemotherapy Agent* OR AB Cancer Chemotherapy Agent* AND TI Cancer Chemotherapy Drug* OR AB Cancer Chemotherapy Drug* AND TI Chemotherapeutic Anticancer Agent* OR AB Chemotherapeutic Anticancer Agent***  **S17 TI Chemotherapeutic Anticancer Drug OR AB Chemotherapeutic Anticancer Drug AND TI Cancer Chemotherapy Drug OR AB Cancer Chemotherapy Drug AND TI Antitumor Agent OR AB Antitumor Agent AND TI Colorectal Surgery OR AB Colorectal Surgery AND TI ( Colon and Rectal Surgery Specialty ) OR AB ( Colon and Rectal Surgery Specialty ) AND TI Colon Surgery Specialty OR AB Colon Surgery Specialty**  **S18 TI Rectal Surgery Specialty OR AB Rectal Surgery Specialty AND TI Postoperative Complication* OR AB Postoperative Complication* AND TI Surgical Wound Infection* OR AB Surgical Wound Infection* AND TI Surgical Site Infection* OR AB Surgical Site Infection* AND TI Postoperative Wound Infection* OR AB Postoperative Wound Infection* AND TI Diarrhea* OR AB Diarrhea***  **S19 TI Nausea OR AB Nausea AND TI ( Postoperative Nausea and Vomiting ) OR AB ( Postoperative Nausea and Vomiting ) AND TI Postoperative Emesis OR AB Postoperative Emesis AND TI Postoperative Vomiting OR AB Postoperative Vomiting AND TI Postoperative Nausea OR AB Postoperative Nausea AND TI Vomiting OR AB Vomiting**  **S20 TI Emesis OR AB Emesis AND TI ( Signs and Symptoms, Digestive ) OR AB ( Signs and Symptoms, Digestive ) AND TI Mucositis OR AB Mucositis AND TI Mucositides OR AB Mucositides AND TI Life Quality OR AB Life Quality AND TI Health-Related Quality Of Life OR AB Health-Related Quality Of Life**  **S21 TI Health Related Quality Of Life OR AB Health Related Quality Of Life AND TI HRQOL OR AB HRQOL AND TI Quality of Life OR AB Quality of Life AND TI Biomarker* OR AB Biomarker* AND TI Biological Marker* OR AB Biological Marker* AND TI Biologic Marker* OR AB Biologic Marker***  **S22 TI Immunologic Marker* OR AB Immunologic Marker* AND TI Immune Marker* OR AB Immune Marker* AND TI Tumor Biomarker* OR AB Tumor Biomarker* AND TI Carcinogen Marker* OR AB Carcinogen Marker* AND TI Biological Tumor Marker* OR AB Biological Tumor Marker* AND TI Cancer Biomarker* OR AB Cancer Biomarker***  **S23 S12 OR S13 OR S14 OR S15 OR S16 OR S17 OR S18 OR S19 OR S20 OR S21 OR S22**  **S24 S8 AND S11 AND S23** |

| Search Strategy | |
| --- | --- |
| ****MedicLatina**** | **S1 MH Colonic Neoplasms OR MH Colorectal Neoplasms OR MH Rectal Neoplasms OR MH Anus Neoplasms OR MH Colorectal Neoplasms, Hereditary Nonpolyposis OR MH Sigmoid Neoplasms OR MH Colitis-Associated Neoplasms**  **S2 TI colonic neoplasms OR AB colonic neoplasms AND TI colon neoplasm OR AB colon neoplasm AND TI cancer of colon OR AB cancer of colon AND TI colon cancer OR AB colon cancer AND TI colonic cancer OR AB colonic cancer AND TI colon adenocarcinoma OR AB colon adenocarcinoma**  **S3 TI colorectal neoplasms OR AB colorectal neoplasms AND TI Colorectal Tumor OR AB Colorectal Tumor AND TI colorectal cancer OR AB colorectal cancer AND TI Colorectal Carcinoma OR AB colorectal carcinoma AND TI rectal neoplasms OR AB rectal neoplasms AND TI Rectum Neoplasm* OR AB Rectum Neoplasm***  **S4 TI rectal tumour OR AB rectal tumour AND TI Cancer of Rectum OR AB Cancer of Rectum AND TI rectum cancer OR AB rectum cancer AND TI rectal cancer OR AB rectal cancer AND TI anal neoplasms OR AB anal neoplasms AND TI Anus Neoplasm* OR AB Anus Neoplasm***  **S5 TI anal cancer OR AB anal cancer AND TI Cancer of Anus OR AB Cancer of Anus AND TI Anus Cancer OR AB Anus Cancer AND TI Familial Nonpolyposis Colon Cancer OR AB Familial Nonpolyposis Colon Cancer AND TI Hereditary Nonpolyposis Colorectal Neoplasms OR AB Hereditary Nonpolyposis Colorectal Neoplasms AND TI lynch syndrome OR AB lynch syndrome**  **S6 TI Hereditary Nonpolyposis Colorectal Cancer OR AB Hereditary Nonpolyposis Colorectal Cancer AND TI Hereditary Nonpolyposis Colon Cancer OR AB Hereditary Nonpolyposis Colon Cancer AND TI Sigmoid Neoplasm* OR AB Sigmoid Neoplasm* AND TI Sigmoid Colon Neoplasm* OR AB Sigmoid Colon Neoplasm* AND TI Sigmoid Cancer OR AB Sigmoid Cancer AND TI Sigmoidal Cancer OR AB Sigmoidal Cancer**  **S7 TI Sigmoid Colon Cancer OR AB Sigmoid Colon Cancer AND TI Cancer of Sigmoid OR AB Cancer of Sigmoid AND TI Colitis Associated Neoplasm* OR AB Colitis Associated Neoplasm* AND TI Colitis Associated Colorectal Cancer* OR AB Colitis Associated Colorectal Cancer* AND TI Colitis Associated Cancer* OR AB Colitis Associated Cancer* AND TI Colitis Associated Colon Cancer* OR AB Colitis Associated Colon Cancer***  **S8 S1 OR S2 OR S3 OR S4 OR S5 OR S6 OR S7**  **S9 MH Prebiotics OR MH Probiotics OR MH Synbiotics OR MH Lactobacillus OR MH Bifidobacterium**  **S10 TI prebiotics OR AB prebiotics AND TI probiotics OR AB Probiotics AND TI synbiotics OR AB Synbiotics AND TI lactobacillus OR AB Lactobacillus AND TI bifidobacterium OR AB bifidobacterium**  **S11 S9 OR S10**  **S12 MH Radiotherapy OR MH Immunotherapy OR MH Immune Checkpoint Inhibitors OR MH Antineoplastic Agents OR MH Colorectal Surgery OR MH Postoperative Complications OR MH Surgical Wound Infection OR MH Diarrhea OR MH Nausea OR MH ( Postoperative Nausea and Vomiting ) OR MH Vomiting**  **S13 MH ( Signs and Symptoms, Digestive ) OR MH Mucositis OR MH Quality of Life OR MH Biomarkers OR MH Biomarkers, Tumor**  **S14 TI Radiotherapy* OR AB Radiotherapy* AND TI Radiation Therapy* OR AB Radiation Therapy* AND TI Radiation Treatment* OR AB Radiation Treatment* AND TI Targeted Radiotherapy* OR AB Targeted Radiotherapy* AND TI Targeted Radiation Therapy* OR AB Targeted Radiation Therapy* AND TI Immunotherapy* OR AB Immunotherapy***  **S15 TI Immune Checkpoint Inhibitor* OR AB Immune Checkpoint Inhibitor* AND TI Immune Checkpoint Blockers OR AB Immune Checkpoint Blockers AND TI Immune Checkpoint Inhibition OR AB Immune Checkpoint Inhibition AND TI CTLA-4 Inhibitor* OR AB CTLA-4 Inhibitor* AND TI PD-L1 Inhibitor* OR AB PD-L1 Inhibitor* AND TI Antineoplastic Agent* OR AB Antineoplastic Agent***  **S16 TI Anticancer Agent* OR AB Anticancer Agent* AND TI Antineoplastic Drug* OR AB Antineoplastic Drug* AND TI Antitumor Drug* OR AB Antitumor Drug* AND TI Cancer Chemotherapy Agent* OR AB Cancer Chemotherapy Agent* AND TI Cancer Chemotherapy Drug* OR AB Cancer Chemotherapy Drug* AND TI Chemotherapeutic Anticancer Agent* OR AB Chemotherapeutic Anticancer Agent***  **S17 TI Chemotherapeutic Anticancer Drug OR AB Chemotherapeutic Anticancer Drug AND TI Cancer Chemotherapy Drug OR AB Cancer Chemotherapy Drug AND TI Antitumor Agent OR AB Antitumor Agent AND TI Colorectal Surgery OR AB Colorectal Surgery AND TI ( Colon and Rectal Surgery Specialty ) OR AB ( Colon and Rectal Surgery Specialty ) AND TI Colon Surgery Specialty OR AB Colon Surgery Specialty**  **S18 TI Rectal Surgery Specialty OR AB Rectal Surgery Specialty AND TI Postoperative Complication* OR AB Postoperative Complication* AND TI Surgical Wound Infection* OR AB Surgical Wound Infection* AND TI Surgical Site Infection* OR AB Surgical Site Infection* AND TI Postoperative Wound Infection* OR AB Postoperative Wound Infection* AND TI Diarrhea* OR AB Diarrhea***  **S19 TI Nausea OR AB Nausea AND TI ( Postoperative Nausea and Vomiting ) OR AB ( Postoperative Nausea and Vomiting ) AND TI Postoperative Emesis OR AB Postoperative Emesis AND TI Postoperative Vomiting OR AB Postoperative Vomiting AND TI Postoperative Nausea OR AB Postoperative Nausea AND TI Vomiting OR AB Vomiting**  **S20 TI Emesis OR AB Emesis AND TI ( Signs and Symptoms, Digestive ) OR AB ( Signs and Symptoms, Digestive ) AND TI Mucositis OR AB Mucositis AND TI Mucositides OR AB Mucositides AND TI Life Quality OR AB Life Quality AND TI Health-Related Quality Of Life OR AB Health-Related Quality Of Life**  **S21 TI Health Related Quality Of Life OR AB Health Related Quality Of Life AND TI HRQOL OR AB HRQOL AND TI Quality of Life OR AB Quality of Life AND TI Biomarker* OR AB Biomarker* AND TI Biological Marker* OR AB Biological Marker* AND TI Biologic Marker* OR AB Biologic Marker***  **S22 TI Immunologic Marker* OR AB Immunologic Marker* AND TI Immune Marker* OR AB Immune Marker* AND TI Tumor Biomarker* OR AB Tumor Biomarker* AND TI Carcinogen Marker* OR AB Carcinogen Marker* AND TI Biological Tumor Marker* OR AB Biological Tumor Marker* AND TI Cancer Biomarker* OR AB Cancer Biomarker***  **S23 S12 OR S13 OR S14 OR S15 OR S16 OR S17 OR S18 OR S19 OR S20 OR S21 OR S22**  **S24 S8 AND S11 AND S23** |

**Appendix 2:** Studies eliminated during the full-text screening.

| Author | Reference |
| --- | --- |
| *Delia, P. 2002* | [82] |
| *Delia, P. 2007* | [83] |
| *Ohigashi, S. 2011* | [84] |
| *Limburg, P. 2011* | [85] |
| *Sun, X. 2012* | [86] |
| *Liu, J. 2014* | [87] |
| *Lee, J. 2014* | [88] |
| *Demers, M. 2014* | [89] |
| *Xu, R. 2016* | [90] |
| *Mansouri-Tehrani, H. 2016* | [91] |
| *Zaharuddin, L. 2019* | [92] |
| *Kaźmierczak-Siedlecka, K. 2020* | [93] |
| *Kwon, H. 2021* | [94] |
| *Carlini, M. 2022* | [95] |

**References**

82. Delia P, Sansotta G, Donato V, Messina G, Frosina P, Pergolizzi S, et al. Prophylaxis of diarrhoea in patients submitted to radiotherapeutic treatment on pelvic district: personal experience. Digestive and Liver Disease. 2002;34:S84–6.

83. Delia P, Sansotta G, Donato V, Frosina G, Messina C, Renzis D, et al. Use of probiotics for prevention of radiation-induced diarrhea. World J Gastroenterol [Internet]. 2007;13:912–5. Available from: www.wjgnet.comhttp://www.wjgnet.com/1007-9327/13/912.asp

84. Ohigashi S, Hoshino Y, Ohde S, Onodera H. Functional outcome, quality of life, and efficacy of probiotics in postoperative patients with colorectal cancer. Surg Today. 2011;41:1200–6.

85. Limburg PJ, Mahoney MR, Ziegler KLA, Sontag SJ, Schoen RE, Benya R, et al. Randomized phase II trial of sulindac, atorvastatin, and prebiotic dietary fiber for colorectal cancer chemoprevention. Cancer Prevention Research. 2011;4:259–69.

86. Sun X-N, Guo Y, Zhu M-L. Effects of probiotics on immune function in postoperative colorectal cancer patients receiving adjuvant chemotherapy. Journal of Practical Oncology. 2012;27:610–2.

87. Liu J, Huang XE. Efficacy of Bifidobacterium tetragenous viable bacteria tablets for cancer patients with functional constipation. Asian Pacific Journal of Cancer Prevention. 2014;15:10241–4.

88. Lee JY, Chu SH, Jeon JY, Lee MK, Park JH, Lee DC, et al. Effects of 12 weeks of probiotic supplementation on quality of life in colorectal cancer survivors: A double-blind, randomized, placebo-controlled trial. Digestive and Liver Disease. 2014;46:1126–32.

89. Demers M, Dagnault A, Desjardins J. A randomized double-blind controlled trial: Impact of probiotics on diarrhea in patients treated with pelvic radiation. Clinical Nutrition. 2014;33:761–7.

90. Xu R, Ding Z, Zhao P, Tang L, Tang X, Xiao S. The effects of early post-operative soluble dietary fiber enteral nutrition for colon cancer. Nutrients. 2016;8.

91. Mansouri-Tehrani HS, Khorasgani MR, Roayaei M. Effects of Probiotics with or without Honey on Radiation-induced Diarrhea. International Journal of Radiation Research. 2016;14:205–13.

92. Zaharuddin L, Mokhtar NM, Muhammad Nawawi KN, Raja Ali RA. A randomized double-blind placebo-controlled trial of probiotics in post-surgical colorectal cancer. BMC Gastroenterol. 2019;19:131.

93. Kaźmierczak-Siedlecka K, Folwarski M, Ruszkowski J, Skonieczna-Żydecka K, Szafrański W, Makarewicz W. Effects of 4 weeks of Lactobacillus plantarum 299v supplementation on nutritional status, enteral nutrition tolerance, and quality of life in cancer patients receiving home enteral nutrition – a double-blind, randomized, and placebo-controlled trial. Eur Rev Med Pharmacol Sci. 2020;24:9684–9604.

94. Kwon H, Chae SH, Jung HJ, Shin HM, Ban OH, Yang J, et al. The effect of probiotics supplementation in postoperative cancer patients: A prospective pilot study. Ann Surg Treat Res. 2021;101:281–90.

95. Carlini M, Grieco M, Spoletini D, Menditto R, Napoleone V, Brachini G, et al. Implementation of the gut microbiota prevents anastomotic leaks in laparoscopic colorectal surgery for cancer:the results of the MIRACLe study. Updates Surg. 2022;74:1253–62.
